# Improved Metal–Semiconductor Interface in Monolayer (1L)-MoS_2_ via Thermally-Driven Ag Filaments as Atomic Scale Edge Contacts Triggered by Selective Annealing Process Using Long Wavelength (1064 nm) Pulsed Laser

**DOI:** 10.1021/acsami.4c20612

**Published:** 2025-04-03

**Authors:** Sumayah-Shakil Wani, Yao-Ren Kuo, K.M.M.D.K. Kimbulapitiya, Ruei-Hong Cyu, Chieh-Ting Chen, Ming-Jin Liu, Huynh-Uyen-Phuong Nguyen, Bushra Rehman, Xin-Rui Liu, Feng-Chuan Chuang, Yen-Fu Lin, Chang-Hong Shen, Po-Wen Chiu, Yu-Lun Chueh

**Affiliations:** 1 Department of Materials Science and Engineering, National Tsing Hua University, Hsinchu 30013, Taiwan; 2 College of Semiconductor Research, National Tsing Hua University, Hsinchu 30013, Taiwan; 3 Department of Physics, National Sun Yat-Sen University, Kaohsiung 80424, Taiwan; 4 Department of Physics, National Chung Hsing University, Taichung 40227, Taiwan; 5 National Applied Research Laboratories, Taiwan Semiconductor Research Institute, Hsinchu 300091, Taiwan; 6 Institute of Electronics Engineering, National Tsing Hua University, Hsinchu 30013, Taiwan; 7 Department of Materials Science and Engineering, Korea University, Seoul 02841, Republic of Korea

**Keywords:** transition metal dichalcogenide, 2D materials, 1064 nm Pulse laser annealing, atomic-scale edge contacts, monolayer (1L)-MoS_2_ FET

## Abstract

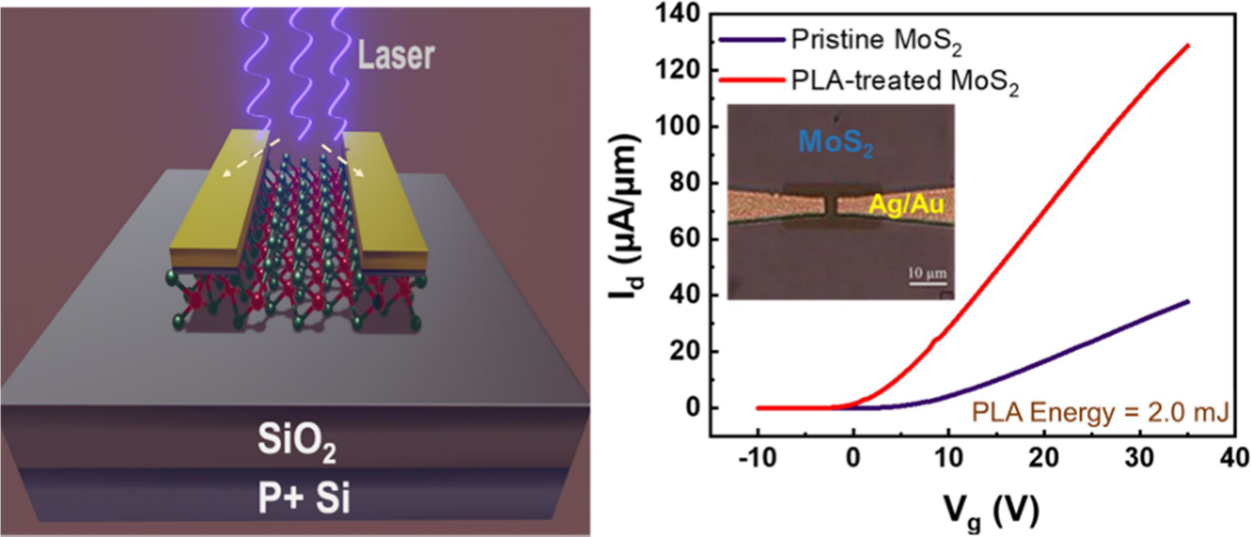

Here, we explore the effectiveness of a pulsed laser annealing (PLA) process to trigger atomic scale edge contacts by Ag filaments in reducing the contact resistance of a MoS_2_ field-effect transistor (FET). Employing a long wavelength (1064 nm) pulsed laser, we anneal monolayer (1L)-MoS_2_ FETs with various metal electrodes, including Ag/Au, Ni/Au, and Cr/Au. A remarkable enhancement in FET performance could be achieved after the PLA treatment. Specifically, Ag/Au-contacted 1L-MoS_2_ FETs after the PLA treatment exhibit a peak field-effect mobility increase from 60 to 135 cm^2^ V^–1^ s^–1^ and an on-current improvement from 40.5 to 96.1 μA at a Vd of 1 V, accompanied by a significant decrease in contact resistance to 0.29 kΩ μm. PLA-treated 1L-MoS_2_ FETs showed a high on/off ratio of 10^7^. TEM analysis provided insight into the mechanism of reduced contact resistance, revealing the thermally driven diffusion of Ag atoms into the 1L-MoS_2_ as Ag filaments to lateral contact with the edge of the 1L-MoS_2_, namely atomic scale edge contacts, as a key contributing factor. Furthermore, our investigation extends to the larger scale CVD-grown 1L-MoS_2_ films, where the PLA treatment demonstrates notable improvements in mobility, on-current, and on–off ratio.

## Introduction

Transition metal dichalcogenides (TMDs) constitute a class of materials characterized by a structured arrangement of transition metal atoms bonded to chalcogen atoms in a layered configuration.^1^ The fundamental compositions of TMDs follow the general formula MX_2_, where the transition metal atoms (M) are situated between layers of chalcogen atoms (X), primarily sulfur (S), selenium (Se), and tellurium (Te). Molybdenum disulfide (MoS_2_), the most widely studied TMD, exhibits a hexagonal lattice structure in which molybdenum atoms are bonded to six surrounding sulfur atoms within each layer.^2^ Weak van der Waals forces between MoS_2_ layers enable easy exfoliation, yielding thin flakes or single-layer structures. Transitioning from an indirect bandgap of 1.2 eV in its bulk form to a direct bandgap of 1.8 eV when reduced to a monolayer, monolayer (1L)-MoS_2_ becomes exceptionally well-suited for optoelectronic applications, while its mechanical flexibility, high thermal conductivity, and catalytic properties further broaden its potential in electronics, energy, and catalysis.^3−6^ Ongoing research explores the diverse applications of MoS_2_ and other TMDs, reflecting their significance in various fields.

The rise of 2D field-effect transistors (2D-FETs) marks a promising trajectory for the evolution of next-generation electronic devices, owing to their distinctive properties and versatile applications.^7,8^ Unlike traditional silicon, 2D materials present atomically flat surfaces with an exceptionally low density of dangling bonds or charge traps. As the realm of 2D material advances, prevalent 2D devices undergo dimensional reduction to optimize their performance, cost, and power consumption. In electronic devices, junctions between metals and semiconductors play a pivotal role, serving as critical elements in device functionality. Notably, these junctions are the crucial link between two-dimensional and three-dimensional domains in devices utilizing two-dimensional materials. In 2D material interfaces, two distinct contact geometries prevail over top and edge contacts. While the formation of the top contact is relatively straightforward, involving the deposition of metals atop 2D materials, the achievement of an optimal edge contact is a significant challenge due to the limited contact area along the edges of 2D materials.^9^ Based on the strength of interfacial interactions, TMD/metal contacts could be delineated into three distinct categories.^10^ (1) In instances where interfacial adhesion is weak and a van der Waals (vdWs) gap exists between the TMD and the metal, an additional tunneling barrier, hampers efficient carrier injection.^9,10^ (2) Conversely, spatial gaps or tunneling barriers become negligible when the interfacial interaction strength is relatively robust, characterized by orbital overlapping between the metal and the underlying TMD. This scenario often leads to unexpected interfacial states induced by metal or defects, such as metal-induced gap states (MIGS), resulting in a Fermi level pinning (FLP) behavior within the TMD bandgap.^11^ It is noteworthy that most 2D devices conform to this contact type.^12^ (3) Finally, in cases where the metal extensively hybridizes with the underlying TMD, inducing metallization and obliterating the TMD bandgap, contact domination occurs within the TMD itself, particularly at an atomically flat and thinner interface.^13^ Consequently, achieving ohmic contact becomes more attainable under such condition. This comprehensive classification elucidates the nuanced interplay between 2D materials and metal contacts, shedding light on strategies for optimizing contact engineering in nanoelectronic devices.

Achieving low electrical contact resistance at metal–semiconductor interfaces is imperative for the creation of high-performance field-effect transistors. However, the presence of a potential energy barrier, commonly referred to as the Schottky barrier, poses a significant challenge, leading to elevated contact resistance levels that could impair device performance and increase energy consumption. Challenges arise due to the Fermi level pinning induced by chemical disorders and defects, increasing Rc and impeding carrier transport between the metal electrode and the semiconductor interface. Conventional deposition of metal electrode processes exacerbated this issue by deviating from the Schottky-Mott rule, forming the high Schottky barrier height (SBH) and the elevated Rc. Therefore, optimizing the contact interface between MoS_2_ and metal electrodes is essential for the enhancement of FET performance. In the realm of engineering 2D contacts, numerous techniques, such as heavily doping semiconductor materials,^14^ introduction of ultrathin dielectric layers, van der Waals gaps,^15,16^ or molecular layers, phase-designed connections,^17^ the utilization of scandium electrodes,^18^ and thermal annealing^7,18−21^ have been explored. However, specific traditional techniques like ion implantation, commonly employed in Si devices for ohmic contacts, prove unsuitable for 2D materials due to lattice damage.^22,23^ While the scandium electrode offers electrical advantages, its limited availability and processing challenges hinder practical applications. Additionally, transforming the semiconducting phase into the metallic phase and locally patterning presents significant hurdles that potentially conflict with standard device fabrication processes. Concerns regarding stability at high manufacturing temperatures and device reliability further complicate phase-controlled connections. Conventional thermal annealing, posing risks to flexible substrates with low thermal budgets and heat-sensitive channels, underscores the necessity for alternative approaches concentrating energy on selective areas to minimize the heat-affected zone.^24^

In this regard, we focus on showcasing the potential for achieving enhanced performance through the implementation of pulsed laser annealing (PLA) treatment to selectively anneal the contact regions of 2D-based FETs. Notably, the PLA treatment replicates the effects of thermal annealing without compromising the integrity of 2D materials, as the impact of the PLA treatment is confined to metals, elevating the temperature of a localized area. Here, we utilize a high-wavelength (1064 nm) pulsed laser to anneal the 1L-MoS_2_-based FET and enhance its performance, with which three different metal/semiconductor interfaces, employing Ag/Au, Ni/Au, and Cr/Au as the metal electrodes, were used. Specifically, for Ag metal as the electrode, the 1L-MoS_2_ exhibits an enhanced peak field-effect mobility from 60 to 135 cm^2^V^–1^s^–1^ with the improved on-current from 40.5 to 96.1 μA at Vd = 1 V because of a decrease in contact resistance to a smaller value of 0.29 KΩ–μm. Analytical tests elucidate the chemical and physical mechanisms underlying the enhanced performance, highlighting the efficacy of the PLA treatment in promoting the diffusion of Ag filaments through the interface of Ag/1L-MoS_2_ as atomic scale edge contacts. These findings hold a significant promise for advancing high-performance transistors based on transition metal dichalcogenides (TMDs) through the pulsed laser annealing treatment on metal contacts while preserving the integrity of the channel and substrate. Collectively, our findings underscore the promise of the PLA treatment as a transformative technology for curtailing contact resistance and elevating the performance benchmarks of FETs predicated on 2D materials like MoS_2_. By delving into the underlying mechanisms governing the PLA treatment and its ramifications on device attributes, this research engenders substantive contributions to the realms of nanoelectronics and materials science.

## Results and Discussion

**Figure 1:**
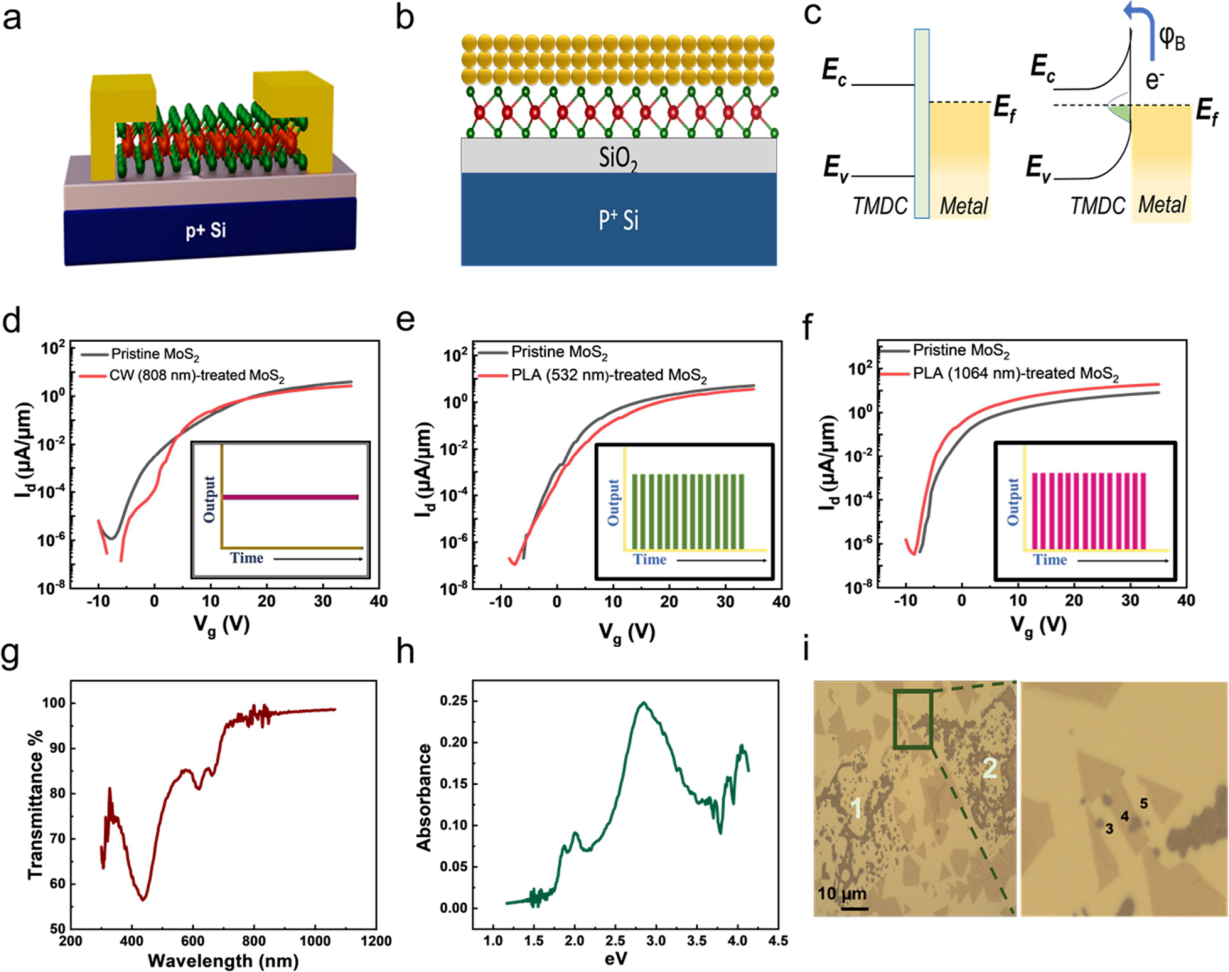
(a) 3D schematic of a back gate monolayer (1L)-MoS_2_ FET. (b) Cross-sectional schematic electrical interface between 1L-MoS_2_ and contact metal. (c) Interface band alignment of van der Waals contact (left) and Schottky-limited contact (rights). Comparative electrical performance after various laser treatments on MoS_2_ FETs, including (d) a continuous wave (CW) laser with the wavelength of 808 nm, (e) a pulsed laser with the wavelength of 532 nm, and (f) a pulsed laser with the wavelength of 1064 nm. Inset (d)-(f) illustrates the output behavior for continuous and pulsed laser. (g) Transmission spectra of the 1L-MoS_2_. (h) Absorbance curve of the 1L-MoS_2_. (i) Optical image of the 1L-MoS_2_ FET with a damaged electrode after the PLA treatment under applied high energy. An enlarged optical image of the 1L-MoS_2_ FET shows no damage to the 1L-MoS_2_ flake except for the areas in contact with the metal electrodes. Positions 1 and 2 represent the damaged electrode pads. Marked position 3 and 5 represent the damaged contact regions, and marked region 4 is the undamaged MoS_2_ channel region after the PLA treatment.

In this study, we aimed to explore the efficacy of the PLA treatment in mitigating contact resistance on FETs based on 2D materials, with a particular focus on monolayer MoS_2_ (1L-MoS_2_), serving as the channel material. The 1L-MoS_2_ was synthesized utilizing the chemical vapor deposition (CVD) technique, yielding triangular domains of varying dimensions, as shown in Figure S1a. The detailed growth of the 1L-MoS_2_ is presented in the material section. The meticulous examination on the structural features of these domains was carried out through precise techniques, such as atomic force microscopy (AFM) and Raman spectroscopy, offering profound insights into their morphology and structural integrity. An AFM imaging, shown in Figure S1b, unveiled an average flake height of approximately ∼0.8 nm. Raman spectroscopy revealed a noticeable frequency discrepancy of about 18 cm^–1^ between E^1^_2g_ and A_1g_ vibrational modes, as corroborated by Figure S1c. Additionally, the consistent photoluminescence (PL) peak position, pinpointed at roughly ∼1.84 eV (∼675 nm), precisely matched the expected response of the 1L-MoS_2_, as evidenced by Figure S 1d. A comprehensive analysis using advanced techniques like SAED and STEM provided crucial insights into the structure and composition of the 1L-MoS_2_. Notably, a STEM image unveiled a nuanced insight into the spatial distribution of Mo and S atoms, leveraging their disparate image contrast levels, as shown in Figure S1e. Note that portraying Mo atoms as relatively bright spots juxtaposed against overlapped pairs of S atoms sheds light on the intricate nanostructure of the 1L-MoS_2_. The clarity and sharpness of the atomic imagery underscored the exceptional crystalline quality of the samples. Complementing this, the SAED pattern captured, as shown in the inset of Figure S1e, depicted a symmetrical hexagonal formation, which is indicative of the underlying hexagonal lattice structure inherent to the 1L-MoS_2_. Furthermore, the investigation revealed a notable presence of S vacancies, as elucidated by the magnified STEM. 1L-MoS_2_ Back-gated FETs were fabricated on a P^+^Si/SiO_2_ (50 nm) substrate to study the electrical performance of the 1L-MoS_2_, as shown in Figure 1a. A 50 nm-thick SiO_2_ gate dielectric layer was deposited using a dry oxidation process. Figure 1b depicts a schematic representation of the 1L-MoS_2_ interface, illustrating how metal contacts function as vital communication links between 1L-MoS_2_ and external circuitry. These contacts significantly influence device performance, impacting parameters, such as drain current,^25^ subthreshold swing, carrier mobility,^26^ power dissipation, etc. The presence of an energy barrier at the junction of metal contact/1L-MoS_2_, arises because of factors like edge dangling bonds, defects, orbital hybridization, or energy differences between the electron affinity of the 1L-MoS_2_ and the work function of metals. This results in the Fermi level pinning, known as the metal-induced gap states (MIGS), as depicted in the right side of Figure 1c.^27−31^ Note that the Fermi-level pinning limits the field effect carrier mobility, preventing access to intrinsic quantum transport phenomena. Additionally, the nature of van der Waals (vdW) contacts imposes constraints on the charge carrier injection into the 1L-MoS_2_ due to the presence of a barrier vdW gap at the interface between the 1L-MoS_2_ and the metal contact, illustrated in the left side of Figure 1c. Understanding the intricacies of the interface between the 1L-MoS_2_ and metal contacts is paramount for optimizing device functionality. To address the inherent challenges associated with these interfaces, we performed the PLA treatment as an alternative to conventional thermal annealing methodologies. The choice of the PLA annealing technique significantly influences the device performance. The PLA treatment emerges as a promising method, offering distinct advantages over the conventional thermal annealing treatment, including rapid heating and cooling cycles, which minimize 1L-MoS_2_ channel material exposure to high temperatures, thereby maintaining crystalline integrity. Its ability to localize heating enables precise annealing in specific regions, depending on different absorption behaviors of materials with respect to the wavelengths of the selected lasers while mitigating damage elsewhere.^32,33^ Moreover, the PLA treatment achieves a higher peak temperature compared to thermal methods, facilitating the enhanced quality of the interface between the 1L-MoS_2_ and metal contacts. In contrast, thermal annealing suffers from the slower processing time and the limitation in reaching an optimal peak temperature. The PLA treatment is also ideal for low thermal budget flexible substrates because of its minimal thermal damage. Figure S2a illustrates the advantages of pulsed laser annealing over conventional thermal annealing. The PLA treatment offers advantages over continuous wave (CW) lasers. One notable drawback is the propensity for CW lasers to induce larger heat-affected zones (HAZ) due to their continuous emission of energy, as illustrated in Figure S2b. This broader thermal footprint could lead to the increase in thermal damage to surrounding areas, potentially compromising device integrity and performance. Overall, its limitations in terms of HAZ size, precision, and adaptability make it less desirable compared to alternative annealing methods such as the PLA treatment. This explains why the performance of the device degrades after the CW laser treatment, as shown in the Figure 1d. The on-current density decreases from the pristine value of 4 to 2.6 μA/μm. In contrast, the PLA treatment could deliver high energy within a short time, resulting in a large instantaneous output and a smaller HAZ. In addition, the high repetition rate of pulses prevents the temperature from dropping to ambient levels before the arrival of next pulse, resulting in the accumulation of heat.^34^ The PLA treatment harnesses high-energy pulses over brief durations, yielding a localized thermal effect.^32,33^ To validate these concepts, the fabricated 1L-MoS_2_ FETs were subjected to the PLA treatment using two different wavelengths of 532 and 1064 nm, respectively. The 1L-MoS2 FETs were subjected to various laser types and wavelengths, all standardized to equivalent energy levels and exposure durations under vacuum conditions, to ensure a more precise and reliable comparison. The 1L-MoS_2_ FET after the PLA treatment with a laser wavelength of 532 nm exhibited performance degradation, as shown in Figure 1e, whereas the PLA treatment on a 1L-MoS_2_ FET with a laser wavelength of 1064 exhibited a significant increase in on-current, as shown in Figure 1f. This disparity could be comprehensively understood by analyzing the transmission and absorption spectra of the 1L-MoS_2_, as illustrated in Figures 1g and 1h. Given that the spot size of the laser is of approximately 1.2 mm, it encompasses not only the source and drain regions but also the channel region. At the laser wavelength of 532 nm, the 1L-MoS_2_ exhibits a relatively low transmittance behavior, indicating that the 1L-MoS_2_ absorbs a significant portion of the incident laser energy. Consequently, when exposed to the laser wavelength of 532 nm, the 1L-MoS_2_ absorbs a substantial amount of the incident energy, leading to localized heating within the material. This localized heating could induce thermal effects within the 1L-MoS_2_ channel region, which ultimately results in the degradation of the electrical performance. However, the PLA treatment using the laser wavelength of 1064 nm confers the advantage of 100% transmittance behavior to the 1L-MoS_2_, exerting its effect solely on the metal part without causing damage to the 1L-MoS_2_ channel region. This explanation is further confirmed by the optical image, as shown in Figures 1i. When the 1L-MoS_2_ FET device was subjected to the PLA treatment with the laser wavelength of 1064 nm at a very high energy for a long duration of ∼1 min, the metal electrodes (marked as 1, 2, 3, and 5) were completely damaged and etched away. However, the 1L-MoS_2_ channel region, marked as 4, remained unaffected.

**Figure 2:**
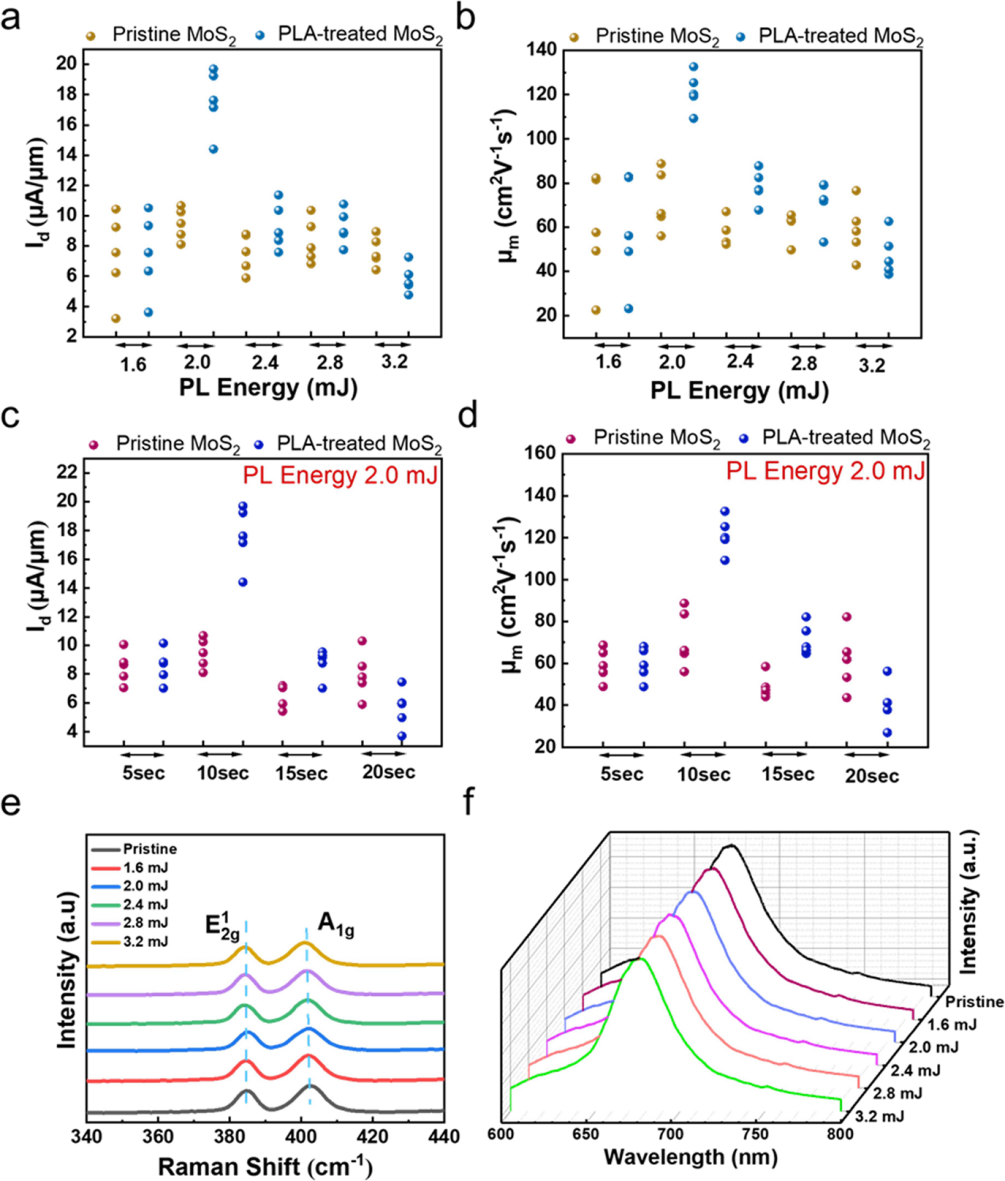
Trends of (a) on current and (b) field-effect mobilities with varying pulse laser energies for a fixed duration of 10 s. Trends of (c) on current and (d) field-effect mobilities with a constant pulse laser energy of 2.0 mJ at different PLA durations for Ag/Au-contacted 1L-MoS_2_ FETs. (e) Raman (f) PL spectra of MoS_2_ channel after different pulse laser energies.

Furthermore, to establish a baseline for the PLA treatment with the laser wavelength of 1064 nm, we conducted experiments to investigate the dependence of the 1L-MoS_2_ FET performance at different laser energy and annealing times. For the comparison, the same devices were analyzed both before and after PLA treatment, with all tests conducted under identical conditions to ensure accuracy. The devices were fabricated with a channel length (*L*_CH_) of 15 μm and a channel width (*W*_CH_) of 5 μm. Figures 2a and 2b represent comparative studies of on-current densities and field-effect mobilities for the pristine and the PLA-treated 1L-MoS_2_ FET at different laser energies, measured in a two-probe configuration, with Ag/Au (10/50 nm) as the contact electrode. All measurements were performed at room temperature in air and were operated under a drain bias voltage (V_d_) of 1 V with gate biases swept from −10 to 35 V. The fabricated 1L-MoS_2_ FETs show a typical n-type behavior, as shown in Figure S3. When the laser energy is insufficiently low, such as 1.6 mJ, it fails to elevate the temperature adequately to influence the contact behavior. Conversely, excessively high laser energy, as that of 3.2 mJ could elevate the annealing temperature to a level that may initiate the deterioration of the contact region. For quantitative assessment, we monitor the increase in laser energy following each PLA treatment. The maximum on-current density with a significant enhancement in mobility was observed at the laser energy of 2.0 mJ. Figures 2c and 2d demonstrate the results of on-current density and corresponding field-effect mobilities for the pristine and the PLA-treated 1L-MoS_2_ FETs at different time durations with the fixed laser energy of 2.0 mJ. Corresponding transfer (I_d_-V_g_) curves were illustrated in Figure S4. As a result, the studies revealed that the duration (time) of 10 s was proved to be favorable in enhancing electrical performance. For a more comprehensive study, we fabricated devices using Ni/Au (10/50 nm) and Cr/Au (5/50 nm) as contact electrodes. Note that Cr serves as an adhesion layer beneath the Au metal electrode for the monolayer MoS_2_ FETs. Both types of devices (Ni/Au- and Cr/Au-contacted) were subjected to the same range of pulse durations at a fixed, optimized laser energy of 2.0 mJ. Similar results were followed by Ni/Au- and Cr/Au-contacted 1L-MoS_2_ FETs (Figure S5), and device performance metrics under the PLA treatment are summarized in Tables S1 and S2. The corresponding Raman and PL spectra of the MoS_2_ channel are illustrated in Figures 2e and 2f, respectively. Results reveal no shift or intensity alteration in two representative Raman peaks (E^1^_2g_, A_1g_) and PL peaks of pristine and PLA-treated 1L-MoS_2_ FETs, indicating that the pristine morphology of the MoS_2_ channel is preserved. Furthermore, AFM was employed to examine the surface morphologies of the 1L-MoS_2_ before and after the PLA treatment, as shown in Figures S6a and S6b, respectively. The improvement in the surface texture of 1L-MoS2 is strikingly evident, with the root-mean-square (RMS) value significantly decreasing from 3.75 to 1.56 nm.

The improvement in surface smoothness observed after the PLA treatment correlates with a substantial decrease in contaminants. This reduction underscores the efficacy of the PLA treatment in mitigating organic contamination on the channels of fabricated MoS_2_ FETs, consequently enhancing both the quality and the performance of these devices. The surface morphology of the 1L-MoS_2_ channel underwent detailed characterization using X-ray photoelectron spectroscopy (XPS) before and after the PLA treatment, as shown in Figures S6c and S6d, respectively. Calibration of all spectral results were conducted utilizing the C 1s peak, maintaining a constant binding energy of approximately 284.6 eV. Remarkably, no shift in the peak was observed, suggesting that the original morphology of the 1L-MoS_2_ channel region remained remarkably intact after the PLA treatment. The Mo 3d_5_/_2_ and Mo 3d_3_/_2_ peaks were consistently measured at 229.25 and 232.35 eV, respectively. Similarly, the S 2p peaks exhibited no shift, with which the S 2p_1_/_2_ and S 2p_3_/_2_ peaks were recorded at 163.4 and 162.25 eV, respectively. The preservation of morphology underscores the robustness of the PLA treatment in maintaining the structural integrity of the 1L-MoS_2_ channel, a crucial aspect for ensuring device reliability and performance in field-effect transistor applications. With the careful selection of parameters such as pulse laser (PL) energy and pulse laser (PL) duration (time), the study facilitated a method to improve the electrical performance of the 1L-MoS_2_ FET. All the devices were PLA treated in a high vacuum of 10^–5^ Torr. The vacuum environment played a crucial role in preventing unwanted oxidative reactions that typically occur in open air. Exposure to oxygen and moisture could cause oxidation of Ag contacts, which significantly degrade the electrical performance of the devices, as shown in Figure S7. In contrast, the high vacuum environment ensured a cleaner process by eliminating reactive gases and contaminants that could interfere with the metal–semiconductor interface. This preservation of the integrity on both the Ag contact region and the 1L-MoS_2_ channel consequently enhanced the overall electrical performance of the device.

**Figure 3:**
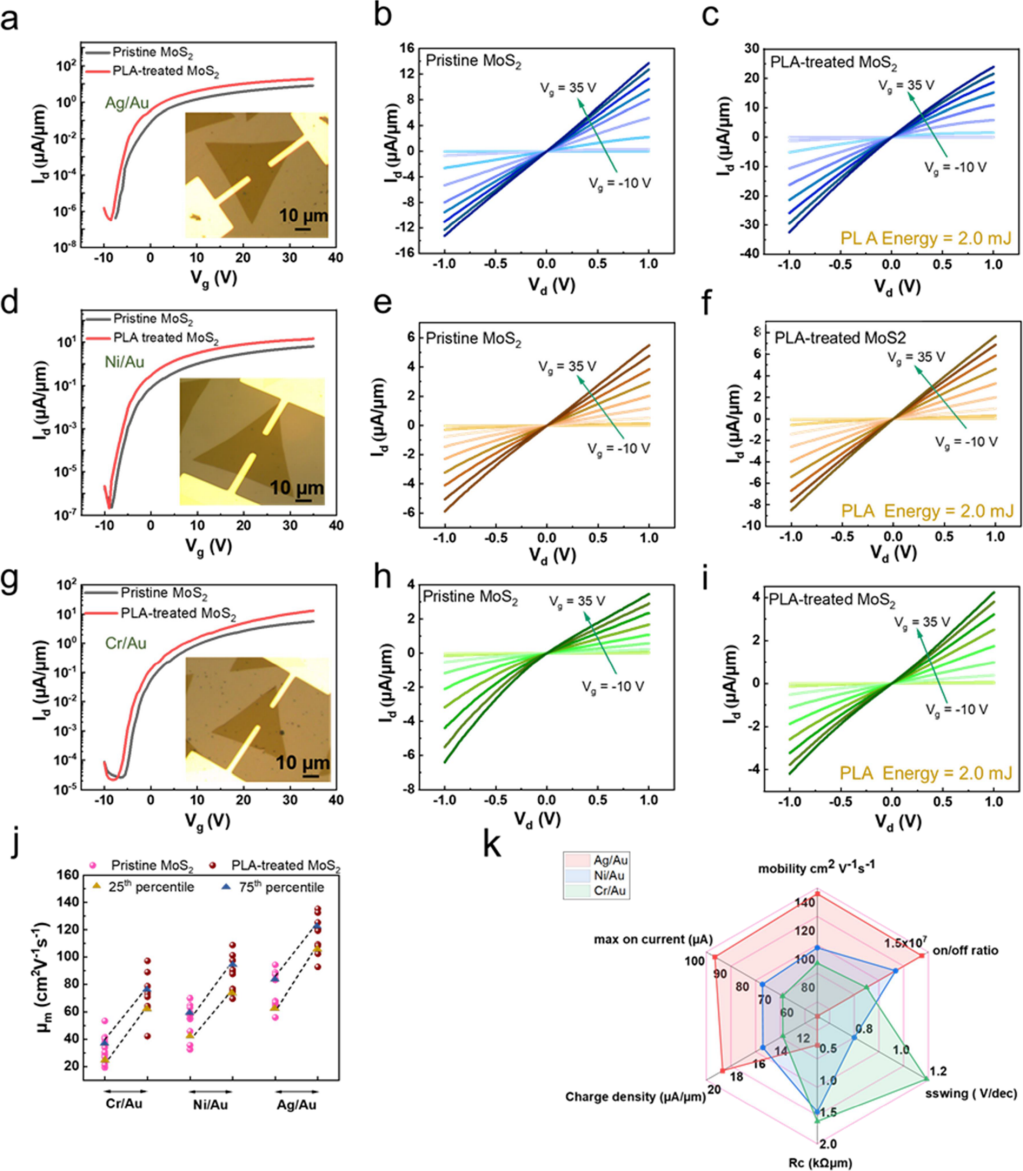
(a) Transfer characteristics of Ag/Au contacted 1L-MoS_2_ FETs before and after the PLA treatment. Output characteristics of 1L-MoS_2_ FETs (b) Pristine MoS_2_ and (c) PLA-treated 1L-MoS_2_ with the Ag/Au metal contact. (d) Transfer characteristics of Ni/Au-contacted 1L-MoS_2_ FETs before and after the PLA treatment. Output characteristics of 1L-MoS_2_ FETs. (e) Pristine 1L-MoS_2_ and (f) PLA-treated 1L-MoS_2_ with the Ni/Au metal contact. (g) Transfer characteristics of Cr/Au-contacted 1L-MoS_2_ FETs before and after the PLA treatment. Output characteristics of 1L-MoS_2_ FETs. (h) Pristine MoS_2_ and (i) PLA-treat 1L-MoS_2_ with the Cr/Au metal contact. Inset displays the OM image of the respective 1L-MoS_2_ FET. (j) Mobility distribution of pristine and PLA-treated 1L-MoS_2_ with different metal contacts (Ag/Au, Ni/Au, and Cr/Au). The graph denotes the 25th and 75th percentile of 10 different 1L-MoS_2_ FET devices. (k) Overview of the electrical performance of 1L-MoS_2_ FET with different electrodes after the PLA treatment.

Figure 3a demonstrates the back-gate performance of the pristine and the PLA-treated 1L-MoS_2_ FETs after the best PLA treatment at 2.0 mJ with 10 s. Figures 3b and 3c show the output characteristics i.e., drain current versus drain voltage (I_d_ -V_d_) of pristine and PLA-treated 1L-MoS_2_ FETs measured at V_g_ = −10 to 35 V with an increment of 5 V at the low-field regime (V_d_ = −1 to 1 V). The transfer characteristic curves (drain current I_d_ vs gate voltage V_d_) demonstrate a significant improvement in its electrical performance, for which the on-current density increases from 8.1 to 19.2 μA/μm. In the PLA treatment, when metal electrodes were exposed to a laser, free electrons within the material absorb the photon energy emitted by the laser. Each successive pulse of the laser deposits energy onto the surface of the material, initiating a localized heating process. However, the rapid succession of pulses prohibits the complete dissipation of the heat before the arrival of the next pulse, leading to a gradual accumulation of thermal energy within the targeted region. This absorption leads to an increase in temperature through interactions between the energized electrons and the lattice structure of the material. By leveraging the controlled accumulation of heat, we were able to orchestrate the conditions necessary for effective annealing. For the Ni/Au-contacted 1L-MoS_2_ FET, the on-current density enhanced from 6.2 to 15 μA/μm (Figures 3d to 3f). A similar trend was observed with the 1L-MoS_2_ FET, having Cr/Au as the contact electrode (Figures 3g to 3i). The on-current density showed significant enhancement from a pristine value of 5.6 to 13 μA/μm for the PLA-treated 1L-MoS_2_ FET. Note that the PLA-treated 1L-MoS_2_ FET revealed good linearity at a low-V_d_ regime, which signifies its transition from Schottky contact to ohmic contact, and a similar trend was also observed for the Ni/Au (Figures 3e and 3f) and Cr/Au as the contact metals in MoS_2_ FETs (Figures 3h and 3i). Figure 3j shows the field-effect mobility (μ) distribution for 20 different devices with different contact metals. The mobility of the PLA-treated 1L-MoS_2_ FETs (marked in golden) is almost double that of the pristine 1L-MoS_2_ FETs (marked in pink). The field-effect mobility (μ) for all devices was extracted from slopes of the transfer curve (I_d_–V_g_) in the linear region and was calculated using the following equation given by , for which L, W, and C are the channel length, the contact width, and the capacitance of the gate oxide. V_d_, I_d_, and V_g_ are drain voltage, drain current, and gate voltage, respectively. Note that 25th and 75th percentile values marked in Figure 3j show a significant increase in mobility for MoS_2_ FETs with different metal contacts. For a large number of 1L-MoS_2_ FETs with the Ag/Au contact metal, the highest mobility achieved after the PLA treatment could be ∼135 cm^2^V^–1^s^–1^. Whereas, for 1L-MoS_2_ FETs with Ni/Au and Cr/Au as contact metals after the PLA treatment, the highest mobility of 108 cm^2^V^–1^s^–1^ and 97 cm^2^V^–1^s^–1^ could be achieved, respectively. To evaluate the impact of PLA treatment on 1L-MoS_2_ FETs using different electrodes, we compared Ag/Au, Ni/Au, and Cr/Au contacts. Key parameters for this comparison are summarized in Figure 3k. Based on our previous discussion, it is evident that the 1L-MoS_2_ FETs using Ag/Au as the contact metal consistently outperformed the others. In addition to high mobility and on-current, the 1L-MoS_2_ FETs using the Ag/Au as the contact metal after the PLA treatment displayed a high on/off current ratio of >10^7^ and a small subthreshold swing (SS) of 0.4 V/decade. To verify that the improved device performance is not solely reliant on the contact shape, we adopted a patterning technique for our CVD-grown MoS_2_ triangles. The 1L-MoS_2_ FETs were fabricated with a channel length (L_CH_) of 2 μm and a channel width (W_CH_) of 5 μm, as illustrated in Figure S8. The on-current density increased from ∼37.8 (pristine) to ∼128.6 μA/μm (after the PLA treatment) with a significant decrease in SS to 0.5 V/dec (after the PLA treatment) from 1.11 V/dec (pristine MoS_2_) (Figures S8a and S8b). The ohmic contact behavior is indicated by the linear relationships of I_d_–V_d_ at the low-field regime after the PLA treatment, as shown in Figures S8c and S8d. The linear behavior in the pristine 1L-MoS_2_ FET could be expected to be due to the superior thermal conductivity of Ag, coupled with the advantageous alignment of work function with the semiconductor properties of MoS_2_, and the performance further enhanced after the PLA treatment. Additionally, the 25th and 75th percentile values marked in Figures S8e and 8f show a significant increase in on/off current ratio and mobility for the patterned MoS_2_ FETs after the PLA treatment. For a large number of MoS_2_ FETs, the highest mobility achieved after the PLA treatment is ∼120 cm^2^V^–1^s^–1^ with an on/off current ratio of ∼10.^7^

**Figure 4:**
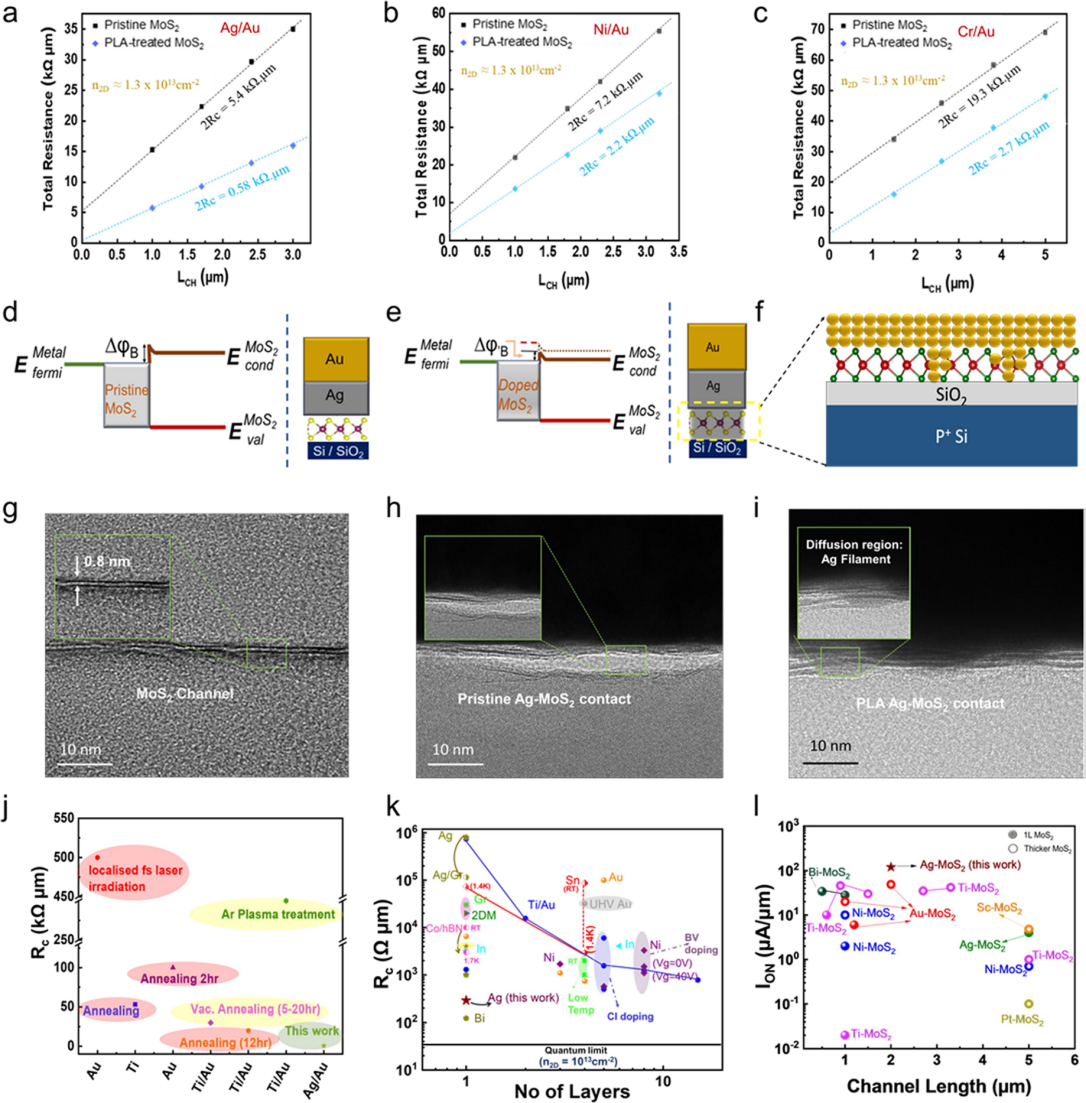
Contact resistance, *R*_c_, obtained using the transmission line method (TLM), demonstrating a dramatic decrease of *R*_c_ after the PLA treatment on 1L-MoS_2_ FET with (a) Ag/Au (b) Ni/Au (c) Cr/Au as metal contacts. (d) Schematic energy-band diagrams of a 1L-MoS_2_ FET with a Schottky barrier at equilibrium. (e) Reduction of Schottky barrier width after the PLA treatment. (f) Schematic of metal-MoS_2_ shows the diffusion of Ag atoms into the 1L-MoS_2_ at the interface after the PLA treatment. Cross-sectional TEM image of (g) a MoS_2_ channel. MoS_2_/Ag interface (h) before and (i) after the PLA treatment. It shows the diffusion of Ag metal into the MoS_2_ at the interface. (j) *R*_c_ reduction induced by various annealing strategies.^37−42^ (k) Scaling of *R*_c_ with different thicknesses of MoS_2_.^1,17,37,39−41,43−56^ (l) On-current density projected as a function of channel lengths (L_CH_) of 1L-MoS_2_ FETs with different contact metals.^1,18,37,43,44,51,53,58^

To assess the impact of the PLA treatment on the contact resistance (Rc) of 1L-MoS_2_ FETs with various metals as contact electrodes, we employed the transfer-length method (TLM). The corresponding OM image of the TLM structure on the patterned MoS_2_ devices is shown in Figure S9. In a two-terminal device, the primary resistance components stem from contact resistance (R_C_) and channel resistance (R_CH_). The total resistance (R_T_) of the device exhibits a linear relationship with the channel length (L_CH_), revealing that the R_C_ (in units of kΩ μm) and the sheet resistance (R_SH_) remain uniform across the device. Consequently, plotting the total resistance of devices against varying L_CH_ enables the depiction of total resistance (R_T_) as a function of L_CH_ under an identical carrier concentration. Hence, by analyzing R_T_ versus L_CH_, the R_C_ values for both pristine and PLA-treated 1L-MoS_2_ FETs with different metals as contact electrodes, such as Ag/Au, Ni/Au, and Cr/Au could be determined at the n_2D_ of 1.3 × 10^13^ cm^–2^, as shown in Figures 4a to 4c. The intercept at L_CH_ = 0 from a linear fit analysis yields the 2R_C_ for the two-terminal MoS_2_ devices. Notably, the satisfactory linear fits observed in the plot of R_T_ normalized by channel width against L_CH_ indicate uniformity in both channel materials and electrical contacts. Utilizing shorter channel lengths could enhance the precision of R_C_ extraction, resulting in data points converging closer to the *y*-axis intersection (2R_C_). It could be seen from Figure 4a that the resistance of the 1L-MoS_2_ FET using the Ag/Au metal as the contact electrode after the PLA treatment decreases from 2.7 to 0.29 kΩ·μm. For the Ni/Au-contacted and Cr/Au-contacted MoS_2_ devices, the contact resistance was reduced from 3.6 and 9.65 kΩ·μm to 1.1 and 1.35 kΩ·μm, respectively, after the PLA treatment, as shown in Figures 4b and 4c. Because Ag/Au-contacted 1L-MoS_2_ FET exhibited the remarkably low R_c.,_ we would focus on the material interface between the Ag metal contact and the 1L-MoS_2_ before and after the PLA treatment in detail. The energy band diagrams of the Ag/Au-contacted MoS_2_ device before and after the PLA treatment are shown in Figures 4d and 4e, respectively. The Fermi level would be pinned at the charge neutrality level (CNL) or S-vacancy level, which is below the conduction band edge with non-negligible SBH, making it challenging to attain a low R_c_ in TMDs by merely utilizing low work function contact metals.^35,36^ The thermionic current would exponentially decrease with the increase in barrier height, making it considerably more difficult for the electrons to transfer from the metal to the MoS_2_. However, the electron injection through the barrier becomes much simpler as the tunneling current begins to outweigh the current through the metal–semiconductor (M-S) junction. When the 1L-MoS_2_ FET is subjected to the PLA treatment, the high energy from the laser could induce localized heating at the interface, promoting thermally driven interdiffusion between the Ag metal and MoS_2_, namely the formation of Ag filaments as edge contacts to the 1L-MoS_2_. In addition, the diffusion of Ag atoms near the contact region of the 1L-MoS_2_ FET could also achieve a n-type doping behavior, which also explains the more negative V_th_ shift from −3.5 V (pristine MoS_2_) to −10 V (PLA treated MoS_2_) (Figure S8b). Figure 4f schematically illustrates the diffusion of Ag filaments into the 1L-MoS_2_ within the contact region after the PLA treatment, with which a penetration behavior of Ag filaments could be formed as the lateral contact to the edge of 1L-MoS_2_, namely atomic scale edge contacts. Results would be validated by cross-sectional TEM images, as shown in Figures 4g to 4i. No deformation occurs at the MoS_2_ channel region after the PLA treatment (Figure 4g). TEM study reveals the intricacies of reducing contact resistance by leveraging the diffusion of Ag through the Ag-MoS_2_ interface via the formation of Ag filaments after the PLA treatment. The formation of Ag filaments in direct contact with the edge of the 1L-MoS_2_ effectively blurred the demarcation between the metal electrode and the 1L-MoS_2_, thereby modifying the electronic properties of the interface region and bolstering electrical conductivity. It is believed that Ag filaments via the thermally driven interdiffusion after the PLA treatment could create many atomic scale edge contacts with the 1L-MoS_2_, enhancing the device performance. This is why the R_c_ values are lower than the best values reported thus far for MoS_2_ devices as plotted to different annealing strategies, as shown in Figure 4j.^37−42^ We also benchmarked our transistor performance with R_c_, a key parameter limiting the scaling of I_ON_ and the on-state current density (I_ON_), a figure of merit in transistor scaling, as shown in Figures 4k and 4l, respectively. The scaling of R_c_ with the thicknesses of MoS_2_ as compared with reported values is shown in Figure 4k.^1,17,37,39−41,43−56^ The solid black line represents the quantum limit of R_c,min_ W = 0.026 (n_2D_)^−0.5^ kΩ·μm calculated at n_2D_ = 1.3 × 10^13^ cm^–2^.^9,57^ The PLA-treated Ag/Au-contacted 1L-MoS_2_ FET (L_CH_ = 2 μm) yields the low R_c_, and the on-current (I_on_), which exceeded the reported values (Figure 4l).^1,18,37,43,44,51,53,58^ As observed from Figure 4k, the Bismuth (Bi)-contacted MoS_2_ shows the lowest R_c_. The study suggests that when a semimetal (Bi) with a near-zero density of states (DOS) at the Fermi level contacts TMDs, gap-state pinning could be inhibited. This inhibition leads to gap-state saturation and, subsequently, low R_c_ at the interface. In contrast, when a normal metal is brought into contact with TMDs, gap-state pinning typically occurs, explaining why Ag/Au-contacted 1L-MoS_2_ has slightly higher but still comparable R_c_.^1,59^ The highest I_ON_ reported by the PLA-treated Ag/Au-contacted 1L-MoS_2_ could be attributed to Rc reduction and the clean MoS_2_ channel. The 1L-MoS_2_ channel has a very strong impact on the electrical performance. Clean 1L-MoS_2_ channel improves on-current by reducing carrier scattering and trapping. With reduced contaminants, charge carriers move more freely, thus increasing the current. This results in higher on-current due to more efficient charge transport and better device performance. Furthermore, no diffusion was observed in the case of the Cr/Au-contacted 1L-MoS2 interface (Figure S10). The absence of diffusion suggests that the Cr/Au metal contact interacts differently with the 1L-MoS_2_ compared to Ag/Au and could be explained by differences in atomic size, bonding properties, or surface energy between Ag/Au and the Cr/Au. One possible reason is that the PLA treatment meticulously removes contaminants and oxides from the interface, enhancing its cleanliness and reducing scattering effects. Further investigation in the future is required to fully elucidate the underlying mechanisms behind this observed discrepancy in diffusion behavior.

**Figure 5:**
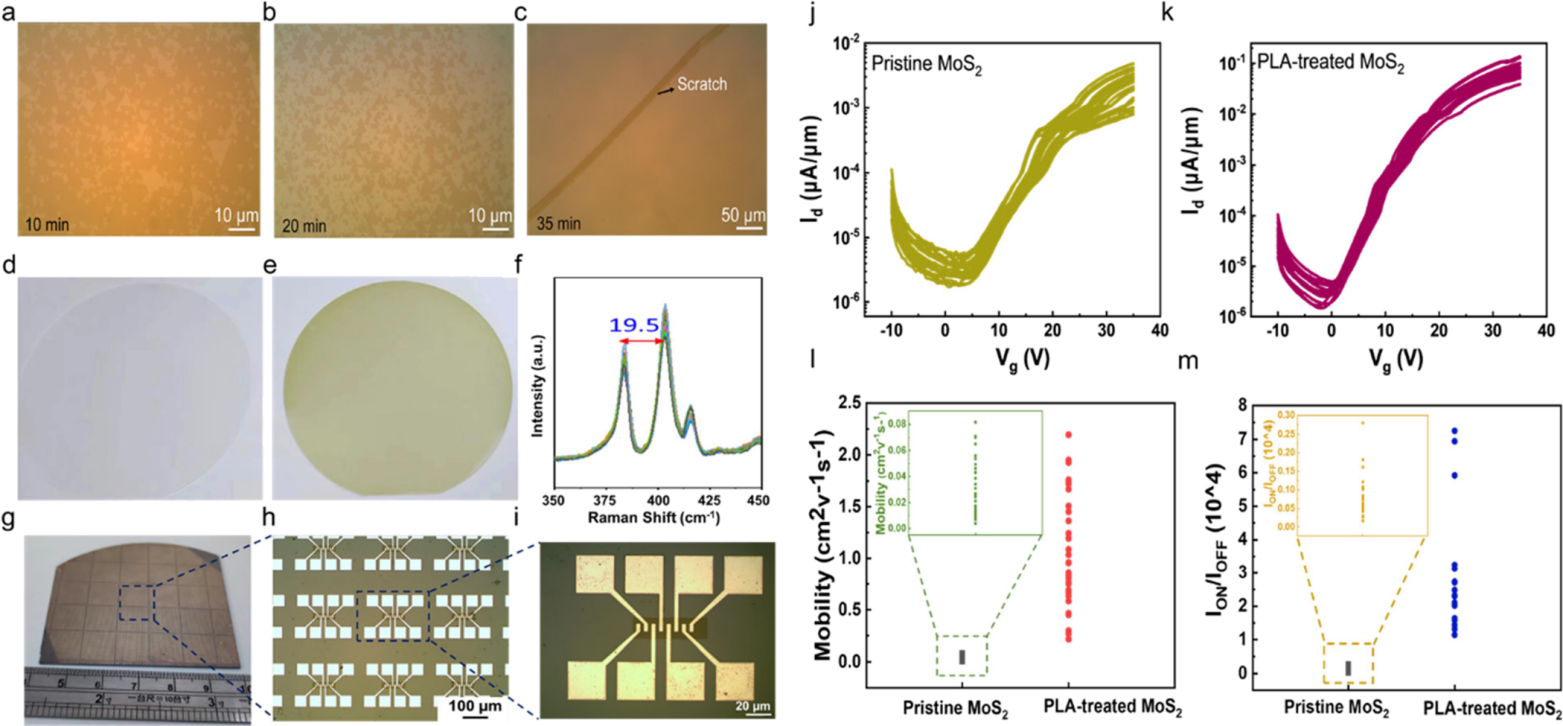
(a–c) Evolution of the triangular-shaped 1L-MoS_2_ flake. As the growth time increases, the individual flakes merge to form a 1L-MoS_2_ film. An image of the clean sapphire substrate (d) before and (e) after the CVD process. (f) Raman spectra of a wafer scale CVD grown 1L-MoS_2_ film taken at different points. (g) Photographic image of the FET arrays fabricated on the 1L-MoS_2_ thin film. (h) Enlarged OM image of the 1L-MoS_2_ FET arrays. (i) OM image of the corresponding 1L-MoS_2_ FETs. Thin film transfer characteristics of the 1L-MoS_2_ FETs. (j) Pristine and (k) PLA-treated 1L-MoS_2_. Overall comparison of trend in arrays of 30 devices with (l) mobility and (m) on/off ratio enhancement after the PLA treatment at a pulse laser energy of 2.0 mJ.

Furthermore, we explored the impact of 1064 nm pulsed laser annealing on a 2-in. 1L-MoS_2_ film grown by the CVD process. Figures 5a-5c illustrate different optical microscopy (OM) images of the 1L-MoS_2_ films grown on sapphire substrates under different growth periods, and detailed procedures regarding film growth and transfer were delineated in the experimental section (see Figures S11). Figure 5d shows an image of a clean 2-in. sapphire wafer before growth, and Figure 5e illustrates an image of a 1L-MoS_2_ film grown on an entire 2-in. sapphire wafer. During the initial 10 min of deposition, MoS_2_ flakes or crystals predominantly form antiparallel islands. This specific orientation is significant as it dictates the subsequent growth behavior of the film. As the deposition time increases to 20 min, the individual flakes grow and begin to merge. Due to the initial lack of a single preferential orientation, the merging of these flakes results in the formation of grain boundaries. As the deposition time increases to 35 min, the entire wafer is covered by the 1L-MoS_2_ film confirmed by Raman spectra, as shown in Figure 5f, where the characteristic E_2g_ and A_1g_ peaks show a 19.5 cm^−1^ separation, consistent with monolayer MoS_2_. A photographic image of the 1L-MoS_2_ FET arrays fabricated on the 1L-MoS_2_ thin film is shown in Figure 5g, and the corresponding enlarged 1L-MoS_2_ FET arrays are shown in Figures 5h and 5i. Subsequent analysis of the electrical characteristics of 30 devices revealed improved performance, as shown in Figures 5j and 5k. Notably, the field-effect mobility surged from 0.07 cm^2^/(V s) to 2 cm^2^/(V s), while the on–off ratio soared from 10^2^ (pristine) to 10^4^ (PLA), as depicted in Figures 5l and 5m. The poor performance of our polycrystalline film before the PLA treatment could be attributed to grain boundaries, which introduce defects and hinder the smooth flow of charge carriers, leading to increased charge carrier scattering and electrical variability compared to single-crystalline films. However, after the PLA treatment, the significant improvement in electrical performance could be achieved.

## Conclusions

In this study, we investigated the potential of the PLA treatment to enhance the performance of FETs based on the 1L-MoS_2_. Achieving low electrical contact resistance at metal–semiconductor interfaces is crucial for realizing high-performance FETs. To address these challenges, we explored the PLA treatment as an alternative annealing technique. Our results demonstrated that the PLA treatment effectively reduces contact resistance and enhances FET performance by leveraging localized heating without compromising the integrity of the 1L-MoS_2_ channel. We fabricated 1L-MoS_2_ FETs with different metal electrodes and subjected them to the PLA treatment, resulting in significant improvements across various performance metrics. For instance, Ag/Au-contacted 1L-MoS_2_ FETs exhibited a peak field-effect mobility increase from 60 cm^2^V^–1^s^–1^ to 135 cm^2^V^–1^s^–1^ and a reduction in contact resistance to 0.29 kΩ μm. Detailed characterization techniques, including Raman spectroscopy, PL, and AFM confirmed the preservation of MoS_2_ morphology. Importantly, the PLA treatment maintained the structural integrity of the 1L-MoS_2_ while enhancing device performance, highlighting its potential as a transformative technology for optimizing metal–semiconductor interfaces in 2D material-based FETs. Moreover, TEM analysis provided insight into the mechanism of reduced contact resistance, revealing the thermally driven diffusion of Ag atoms into the 1L-MoS_2_ as Ag filaments to laterally contact with the edge of the 1L-MoS_2_, namely atomic scale edge contacts, as a key contributing factor. Our findings contribute to advancing the field of nanoelectronics and materials science, offering insights into the development of high-performance electronic devices based on 2D materials like MoS_2_.

## Experimental Section

### Configuration of Pulsed Laser Annealing System

We utilized a pulsed neodymium-doped yttrium aluminum garnet (Nd:YAG) laser, which operates in Q-switching mode. This laser emits at a wavelength of 1064 nm and a frequency of 1000 Hz. The laser beam, with a diameter of 1.2 mm, was focused at a working distance of 55 mm. Precise control over the laser annealing time was achieved using a mechanical shutter. The pulsed laser annealing (PLA) treatment was carried out in a vacuum of approximately ∼10^5^ Torr. For this study, a point scan method was employed, whereas line scans required manual movement of the base plate or substrate holder. To ensure stable performance, the laser underwent a warm-up period of 15 min before the experimental session.

### Growth of Single Crystalline MoS_2_ Triangular Monolayer

Molybdenum(VI) oxide (MoO_3_) powder (Alfa Aesar, 99.999%) and sulfur powder (Aldrich 99.5–100.5%) were used as precursors in the chemical vapor deposition (CVD) growth of the MoS_2_ triangular monolayer on a sapphire substrate. To ensure a successful deposition, the sapphire substrate was ultrasonicated in acetone, isopropanol, and deionized water for 10 min prior to deposition. The MoO_3_ (2 mg) and sapphire substrate were placed separately in tiny tubes in the middle of the quartz tube, while the sulfur powder was inserted upstream. Initially, tube was pumped to a base pressure of 200 mTorr, before introducing argon (Ar, 30 sccm) to raise the working pressure to 560 Torr. Subsequently, the furnace was ramped up to 850 °C and maintained at this temperature for 40 min, with a continuous flow of 30 sccm of argon. Meanwhile, the sulfur source was heated independently using an external heating belt at a temperature of 180 °C. After 10 min of growth, the furnace was cooled to below 100 °C, and the samples were removed from the furnace. The controlled process employed herein facilitated the successful growth of a single crystalline MoS_2_ triangular monolayer on the sapphire substrate.

### Growth of 2-in. Wafer Polycrystalline 1L-MoS_2_ Film

The growth was performed in a low-pressure CVD system equipped with a small inner tube inside the 2-in. size tube (Figure S11a). The small tube was loaded with 50 mg of MoO_3_ (Alfa Aesar, 99.999%) and flowed with Ar/O_2_ (80/3 sccm), and a silica crucible containing 1 g of sulfur (Aldrich, 99.5–100.5%) was positioned at the upstream of the substrate and flowed with Ar (150 sccm). Prior to growth, sapphire wafers were annealed at 1050 °C for 3 h in an oxygen atmosphere to create atomically flat surface for subsequent *MoS*_*2*_ growth. A typical growth of film lasts for ∼35 min at ∼3 Torr.

### Transfer Method of 1L-MoS*_2_* to a SiO_2_/P^+^-Si Substrates

#### For 1L-MoS_2_ Triangle Flakes

The transfer process of the two-dimensional (2D) material involved meticulous three-step processes, comprising separation, fishing up, and removal. Initially, a layer of poly(methyl methacrylate) (PMMA 950 A4) was spin-coated onto the freshly as-grown 2D material at 800 rpm for 10 s, followed by 2000 rpm for 30 s to establish a robust supporting layer. The higher rotational speed in the latter phase facilitated the attainment of a uniform and smooth PMMA layer. Subsequently, the edges of the film were delicately scraped using tweezers to create a narrow gap between the PMMA/2D material film and the original substrate, ensuring minimal damage to the film. This gap played a pivotal role in the subsequent separation phase. A diluted ammonia solution (NH_4_OH:DI water = 1:5) was then employed to separate the edges effectively. In the fishing up stage, the film was meticulously peeled off and cleansed with deionized (DI) water. The delaminated PMMA/2D material film was gently maneuvered into a low-polarity solution comprising a blend of alcohol and DI water, facilitated by a glass slide. A pristine target substrate was utilized to retrieve the floating delaminated stack of PMMA/2D material. In the removal step, any residual droplets trapped between the film and the target substrate were evaporated by heating the substrate to 70 °C for 20 min, ensuring enhanced adhesion of the 2D material to the target substrate. Following baking, the entire substrate underwent immersion in an acetone solution at room temperature for 30 min to dissolve the PMMA layer effectively. Subsequently, thorough cleaning with isopropyl alcohol (IPA) and DI water was conducted to eliminate any remaining residues and contaminants.

#### For a 2 in. 1L-MoS_2_ Thin Film

A layer of poly(methyl methacrylate) (PMMA 950 A8) was spin-coated onto the freshly grown 2D material at 800 rpm for 20 s, followed by 2000 rpm for 40 s to establish a robust supporting layer. A similar strategy was applied for the transfer of the MoS_2_ thin film, with the exception of utilizing a 2 M KOH solution instead of the NH_4_OH:DI solution. The use of KOH solution facilitated smooth and facile delamination of the PMMA/MoS_2_ film (Figure S11 b).

### Fabrication of MoS_2_ Field Effect Transistors

The successfully transferred *MoS*_*2*_ triangles and thin films onto the p+Si/SiO_2_ (50 nm) were further utilized for the back-gate FET fabrication. Direct Light Patterning (DLP) was used to define the channel and the source/drain with S18134 photoresist. After the exposure, the patterns were developed using the AD-10 developer. For the thin MoS_2_ film case, the transferred *MoS*_*2*_ film was first patterned with the help of the hard mask using UV lithography. Then, oxygen plasma dry etching (150 W, 8 s) was employed to etch away the unwanted film, leaving behind the desired pattern. Again, UV lithography was employed for the source (S) and drain (D) electrodes. Metallization was implemented by e-beam evaporation at a deposition rate of 0.3 Å/s, at ∼10^–6^ Torr. The final step is the lift off process, during which the device was immersed in a PG Remover to remove the photoresist, followed by rinsing with acetone, IPA, and DI water.

### Measurements and Characterization

Various techniques were employed to characterize the morphology, size, thickness, and uniformity of MoS_2_, using optical microscopy (OM), high-resolution transmission electron microscopy (HRTEM) (JEOL, JEM-F200, 200 kV), atomic force microscopy (AFM) (Bruker, Dimension Icon), Raman, and PL spectroscopy (HORIBA, LabRAM HR800 with a 532 nm laser). To study MoS_2_ surface before and after the PLA treatment, X-ray photoelectron spectroscopy (XPS) surface analysis was performed. Raman and PL measurements were performed with a 532 nm laser at a power of 50 mW, and the Si peak at 520 cm^–1^ was used as the standard reference peak. Cross-sectional images of the transferred MoS_2_ were obtained using HRTEM (JEOL, JEM-F200, 200 kV) at 200 keV. The electrical properties of the devices were measured using a semiconductor parameter analyzer (Agilent, B1500A)

## Supporting Information Available

Tables of key performance metrics of Ni/Au-contacted and Cr/Au-contacted 1L-MoS_2_ FETs at different durations at an optimized pulse laser energy of 2.0 mJ; material characterizations of CVD grown 1L-MoS_2_; illustrative comparison between conventional thermal and laser annealing processes; transfer curves on drain current with different gate voltages for Ag/Au-contacted pristine and PLA-treated 1L-MoS_2_ FETs at different laser energies; transfer curves on drain current to gate voltages for Ag/Au-contacted pristine and PLA treated 1L-MoS_2_ FETs at pulsed laser energy of 2 mJ for different time durations; transfer curves on drain current to gate voltages for Ni/Au-contacted pristine, Cr/Au-contacted pristine and PLA-treated 1L-MoS_2_ FETs at a fixed pulsed laser energy of 2.0 mJ at different time durations; AFM image analysis of pristine MoS_2_ and PLA-treated MoS_2_; PLA-treated 1L-MoS_2_ flake; XPS spectra comparing pristine and PLA-treated 1L-MoS_2_; transfer characteristics of Ag/Au-contacted 1L-MoS_2_ FET before and after PLA treatment performed in vacuum (∼10^–5^ Torr) and air conditions; transfer characteristics of Ag/Au-contacted patterned 1L-MoS_2_ FETs before and after the PLA treatment at PL energy of 2.0 mJ at *V*_d_ = 1 V; OM image of TLM structure on a patterned 1L-MoS_2_; A cross-sectional TEM image of MoS_2_/Cr interface after the PLA treatment at PL energy 2.0 mJ; and schematic illustration of the CVD growth process on a 2-in. 1L-MoS_2_ thin film (PDF)
