# Supplementary material for: Improved Metal–Semiconductor Interface in Monolayer (1L)-MoS2 via Thermally-Driven Ag Filaments as Atomic Scale Edge Contacts Triggered by Selective Annealing Process Using Long Wavelength (1064 nm) Pulsed Laser
Source: ACS Appl Mater Interfaces. 2025 Apr 3;17(15):23209–21. doi: 10.1021/acsami.4c20612 (PMC12012742; doi:10.1021/acsami.4c20612)
Supplement: Supplementary file 1 — am4c20612_si_001.pdf [file am4c20612_si_001.pdf]

Supporting Information

Improved Metal-Semiconductor Interface in Monolayer (1L)-MoS_2 via Thermally-Driven Ag Filaments as Atomic Scale Edge Contacts Triggered by Selective Annealing Process Using Long Wavelength (1064 nm) Pulsed Laser

Sumayah-Shakil Wani[1], Yao-Ren Kuo[1], K.M.M.D.K. Kimbulapitiya[1, 3], Ruei-Hong Cyu[1], Chieh-Ting Chen[1], Ming-Jin Liu[1], Huynh-Uyen-Phuong Nguyen[1], Bushra Rehman[1], Xin-Rui Liu[1], Feng-Chuan Chuang[3], Yen-Fu Lin[4], Chang-Hong Shen[5], Po-Wen Chiu[2, 6], and Yu-Lun Chueh[1, 2, 3, 7]*

[1]Department of Materials Science and Engineering, National Tsing Hua University, Hsinchu 30013, Taiwan

[2]College of Semiconductor Research, National Tsing Hua University, Hsinchu 30013, Taiwan

[3]Department of Physics, National Sun Yat-Sen University, Kaohsiung, 80424, Taiwan

[4]Department of Physics, National Chung Hsing University, Taichung, 40227, Taiwan

[5]National Applied Research Laboratories, Taiwan Semiconductor Research Institute, Hsinchu, 300091, Taiwan

[6]Institute of Electronics Engineering, National Tsing Hua University, Hsinchu 30013, Taiwan

[7]Department of Materials Science and Engineering, Korea University, Seoul 02841, Republic of Korea.

*E-mail: ylchueh@mx.nthu.edu.tw

Table S1: Key performance metrics of Ni/Au-contacted 1L-MoS_2 FETs at different durations at an optimized Pulse laser energy of 2.0 mJ.

Ni/Au	5sec		15sec		20sec	
	Pristine	PLA	Pristine	PLA	Pristine	PLA
On current (μA)	29.9	30	28	43.5	35.8	24.4
Mobility ($cm^2V^{-1}s^{-1}$)	43	43	44.6	65	54	37.6
On/Off ratio	$\sim10^7$	$\sim10^7$	$\sim10^6$	$\sim10^7$	$\sim10^7$	$\sim10^5$
Current density (μA/μm)	5.98	6	5.6	8.7	7.17	4.8

Table S2: Key performance metrics of Cr/Au-contacted 1L-MoS_2 FETs at different durations at an optimized Pulse laser energy of 2.0 mJ.

CrAu	5sec		15sec		20sec	
	Pristine	PLA	Pristine	PLA	Pristine	PLA
On current (μA)	18.5	18.8	19.8	29.5	18.2	11.1
Mobility ($cm^2V^{-1}s^{-1}$)	24.8	25	28.4	40.6	26.3	17.7
On/Off ratio	$\sim10^7$	$\sim10^7$	$\sim10^7$	$\sim10^7$	$\sim10^7$	$\sim10^5$
Current density (μA/μm)	3.7	3.76	3.96	5.9	3.64	2.22

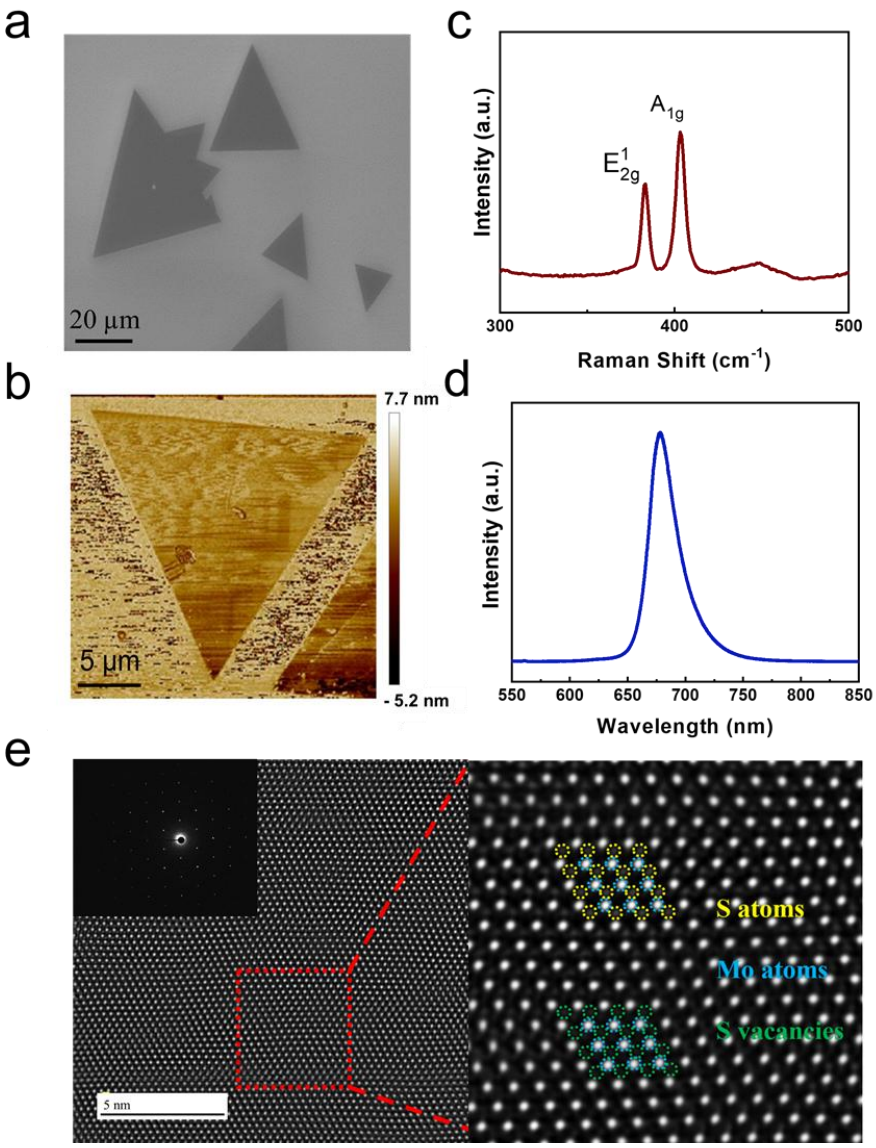

Figure S1 (a) A SEM image of CVD grown 1L-MoS_2. (b) An AFM image of the as-grown 1L-MoS_2. (c) Raman and (d) PL spectra of the as-grown 1L-MoS_2. (e) High-resolution STEM images of an obtained 1L-MoS_2 grown by CVD. The inset shows a SAED pattern of the 1L-MoS_2. (Right) A magnified STEM image shows S Vacancies.

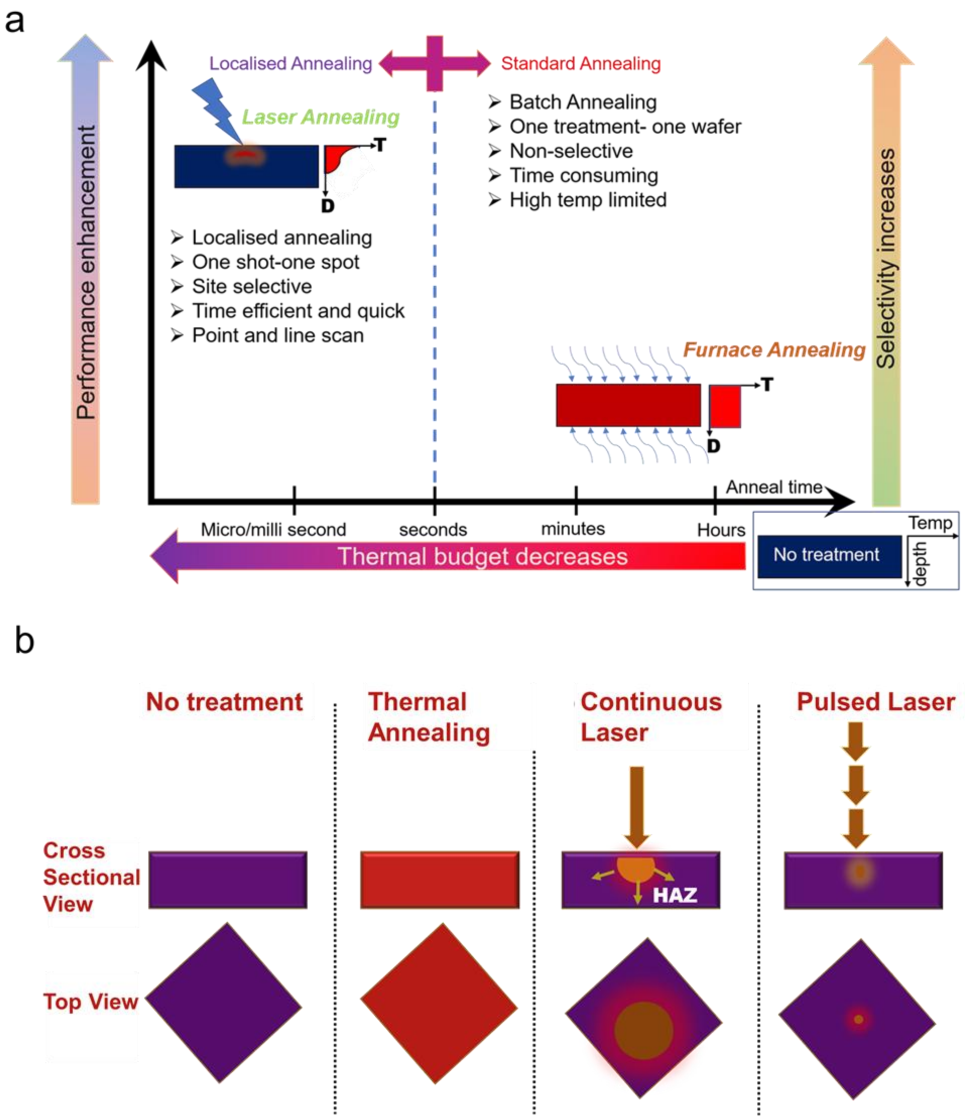

Figure S2 (a) Illustrative comparison between conventional thermal and laser annealing processes. (b) A schematic diagram demonstrates the effect of thermal, continuous wave, and pulsed laser annealing processes.

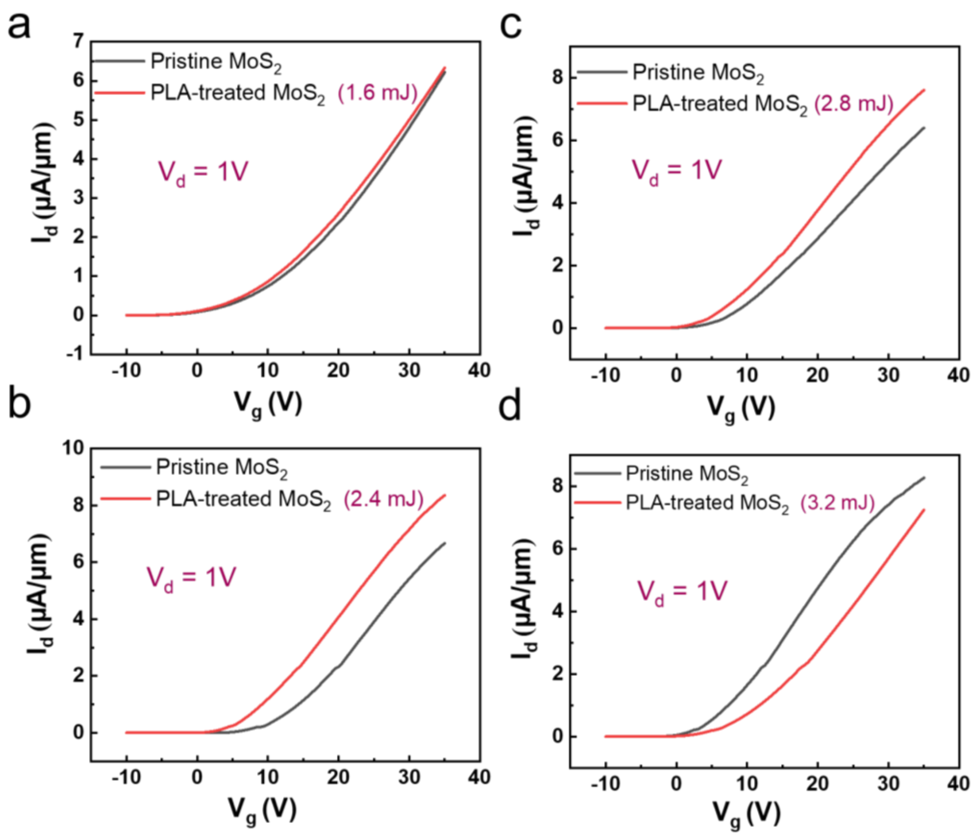

Figure S3 Transfer curves on drain current with different gate voltages, (I_d-V_g), for Ag/Au-contacted pristine and PLA-treated 1L-MoS_2 FETs at different laser energies of (a) 1.6, (b) 2.4, (c) 2.8, and (d) 3.2 mJ, respectively.

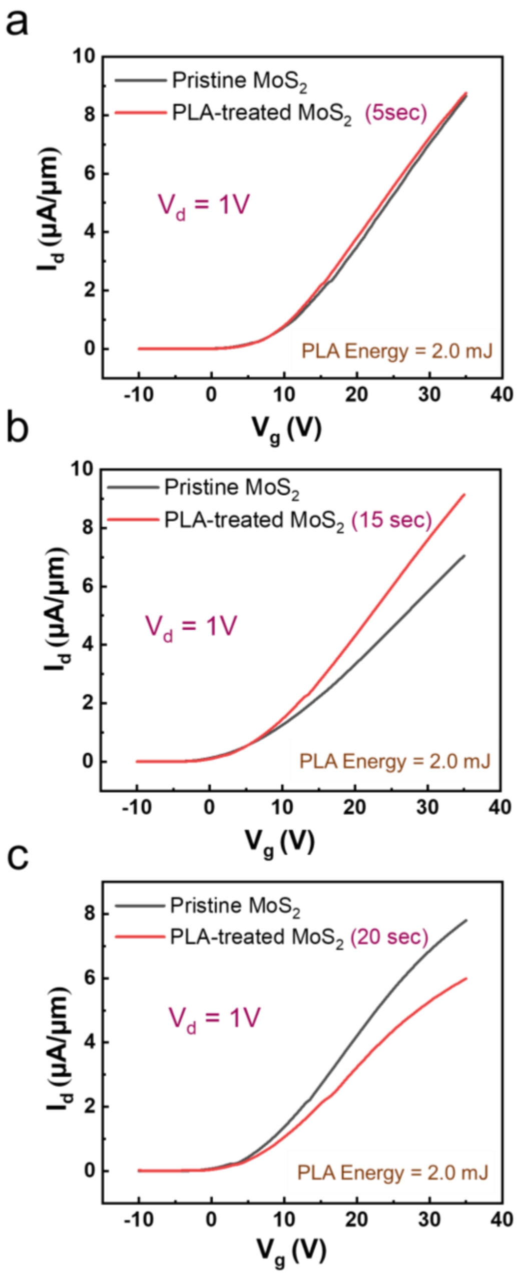

Figure S4 Transfer curves on drain current to gate voltages, (I_d-V_g), for Ag/Au-contacted pristine and PLA treated 1L-MoS_2 FETs at pulsed laser energy of 2 mJ for different time durations of (a) 5, (b) 15, and (c) 20 sec, respectively.

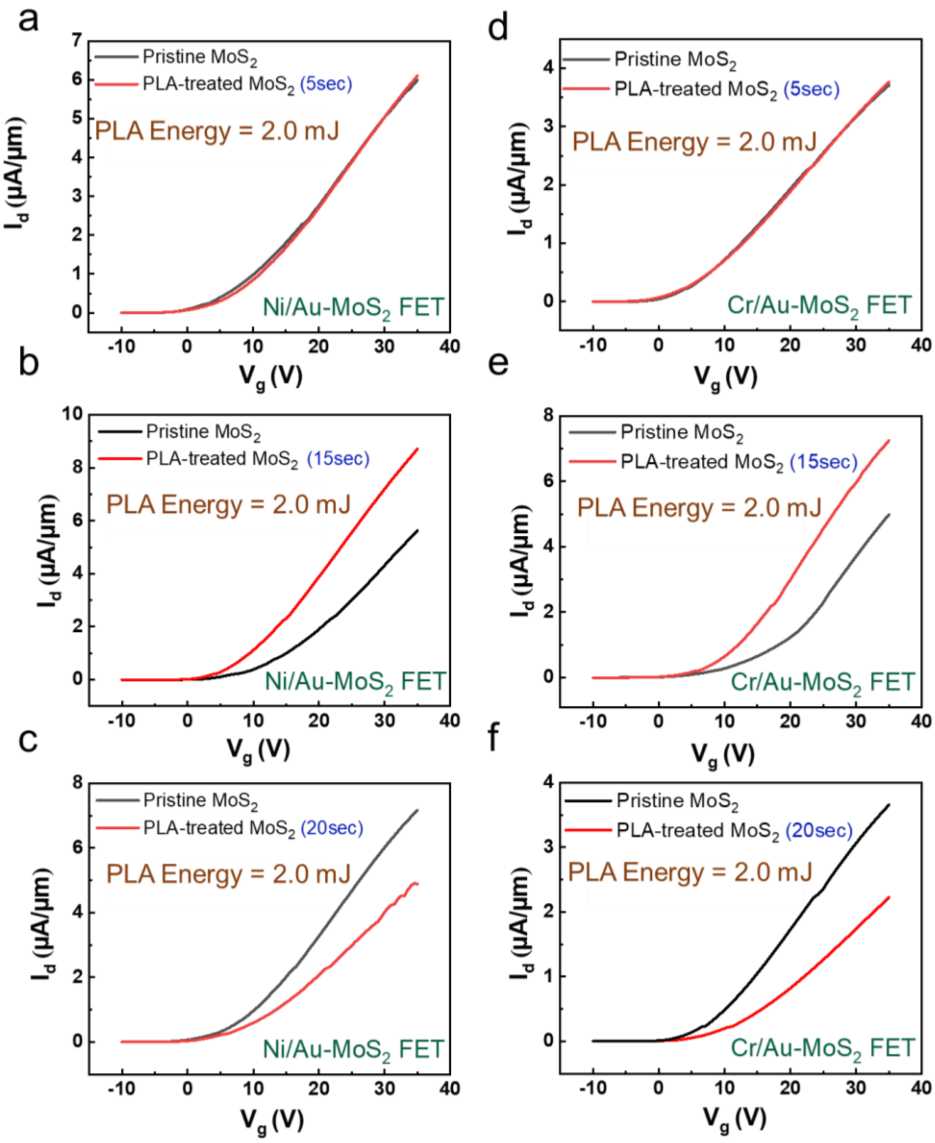

Figure S5 Transfer curves on drain current to gate voltages, (I_d -V_g), for Ni/Au-contacted pristine and PLA-treated 1L-MoS_2 FETs at a fixed pulsed laser energy of 2.0 mJ at different time durations: (a) 5, (b) 15, and (c) 20 sec. Transfer curves on drain current to gate voltages, (I_d -V_g), for Cr/Au-contacted pristine and PLA-treated 1L-MoS_2 FETs at a pulsed laser energy of 2.0 mJ at different time durations of (d) 5, (e) 15, and (f) 20 sec, respectively.

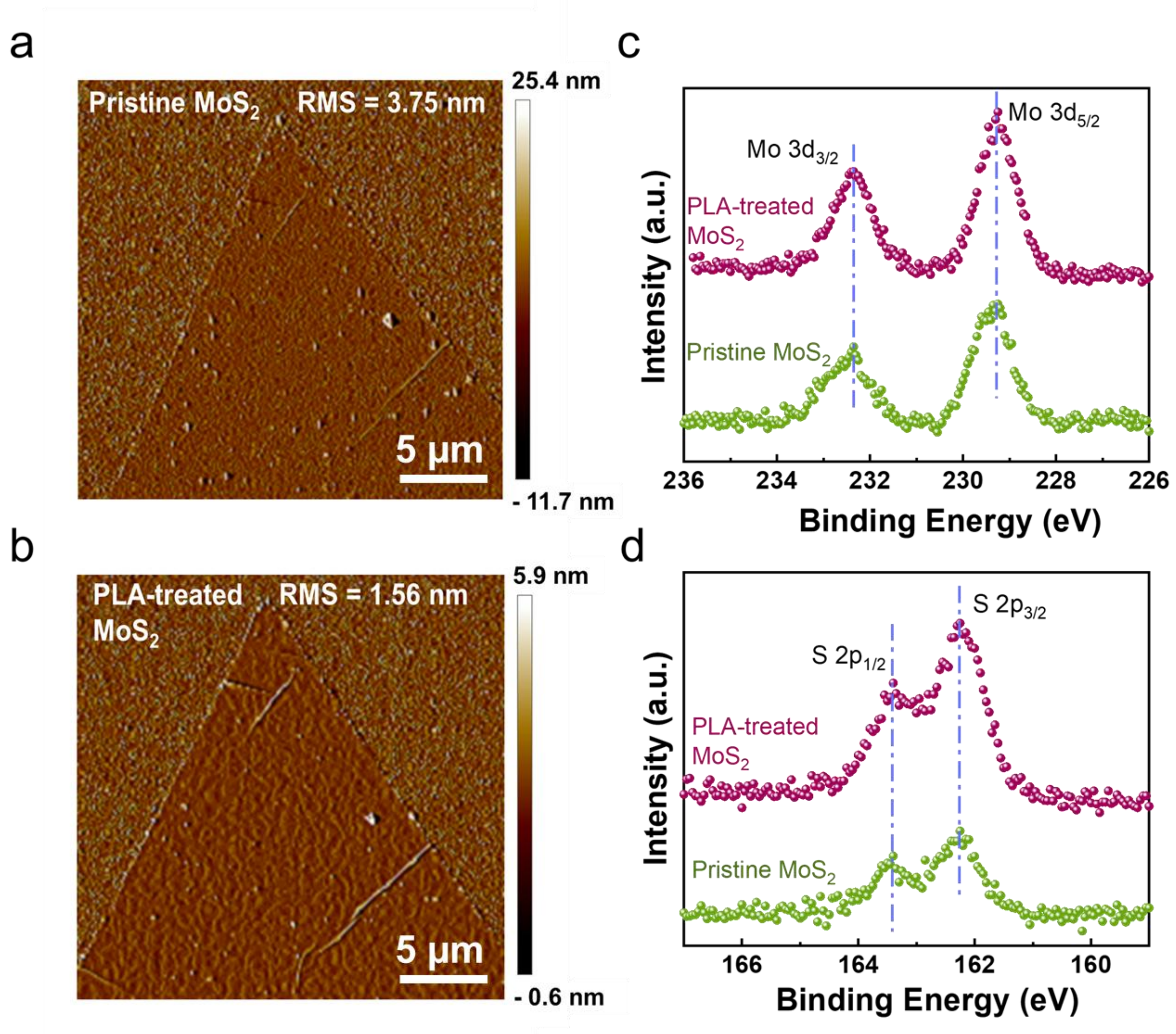

Figure S6 AFM images of (a) Pristine MoS_2 (b) PLA-treated MoS_2. PLA-treated 1L-MoS_2 flake shows significantly reduced roughness. (c)-(d) XPS spectra comparing pristine and PLA-treated 1L-MoS_2.

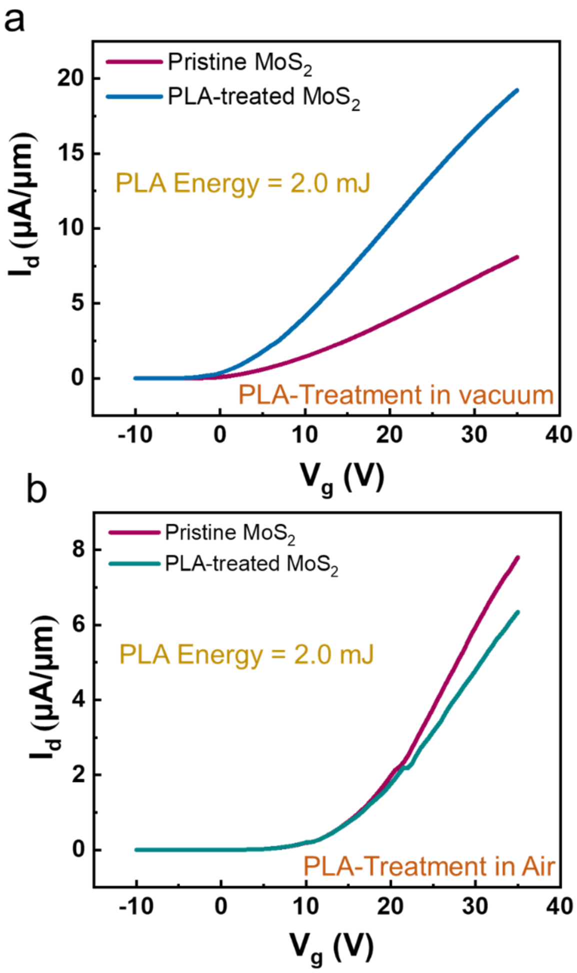

Figure S7 Transfer characteristics of Ag/Au-contacted 1L-MoS_2 FET before and after PLA treatment performed in (a) vacuum (~10^{-5} torr) and (b) air conditions.

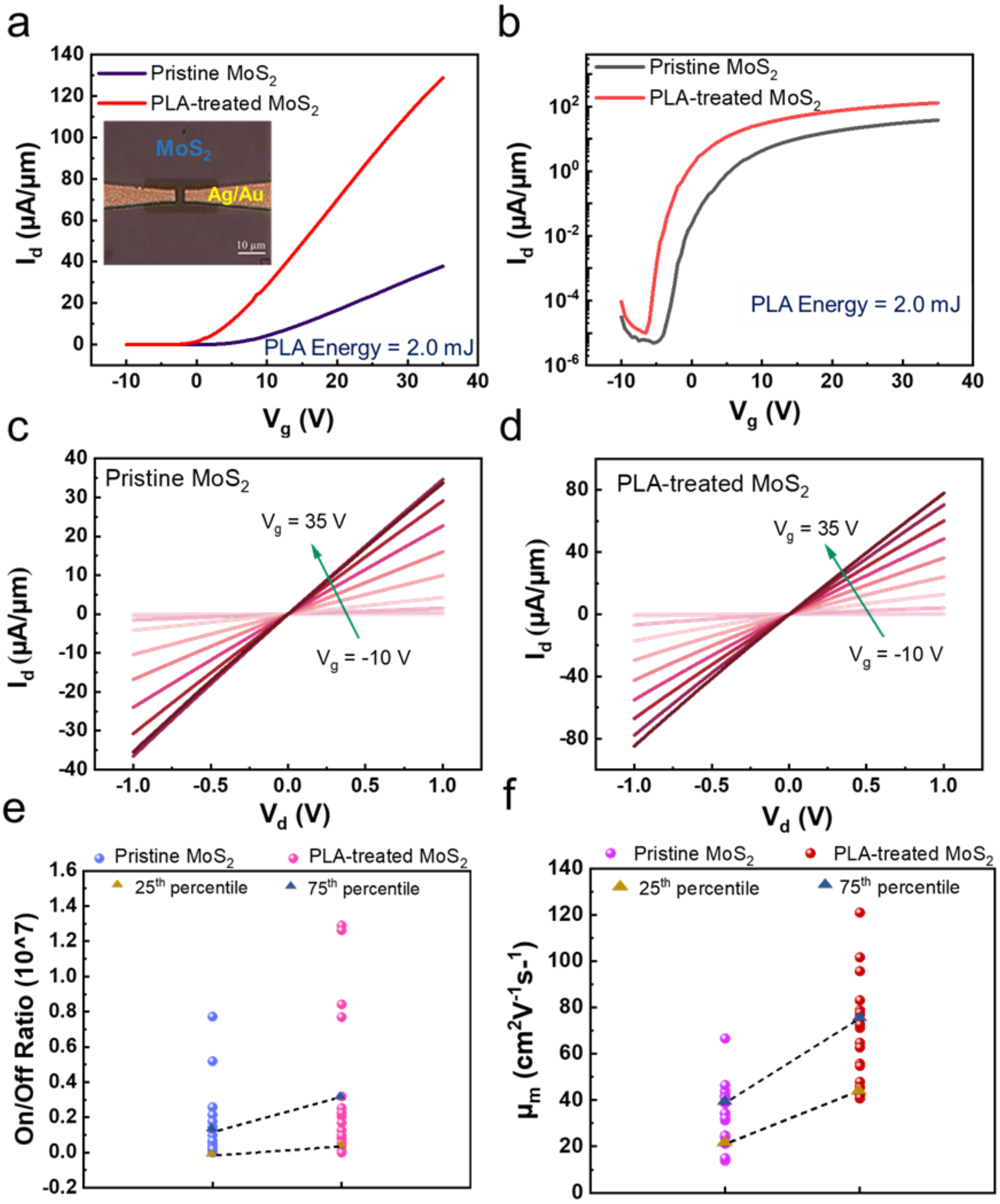

Figure S8 Transfer characteristics of Ag/Au-contacted patterned 1L-MoS_2 FETs before and after the PLA treatment at PL energy of 2.0 mJ at $V_d = 1$ V in (a) linear scale. (The inset shows the OM image of the patterned 1L-MoS_2. Scale bar: 10 µm) and (b) log scale. Output characteristics of 1L-MoS_2 FETs (c) Pristine 1L-MoS_2 and (d) PLA-treated 1L-MoS_2 (e) On/Off ratios and (f) Mobility distribution of pristine and PLA-treated 1L-MoS_2 FETs. The graph denotes the 25th and 75th percentile of 20 different FETs.

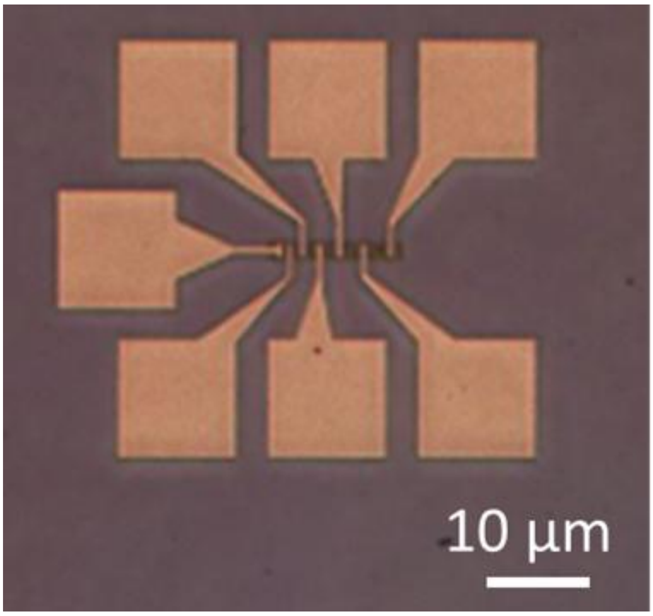

Figure S9 An OM image of TLM structure on a patterned 1L-MoS_2.

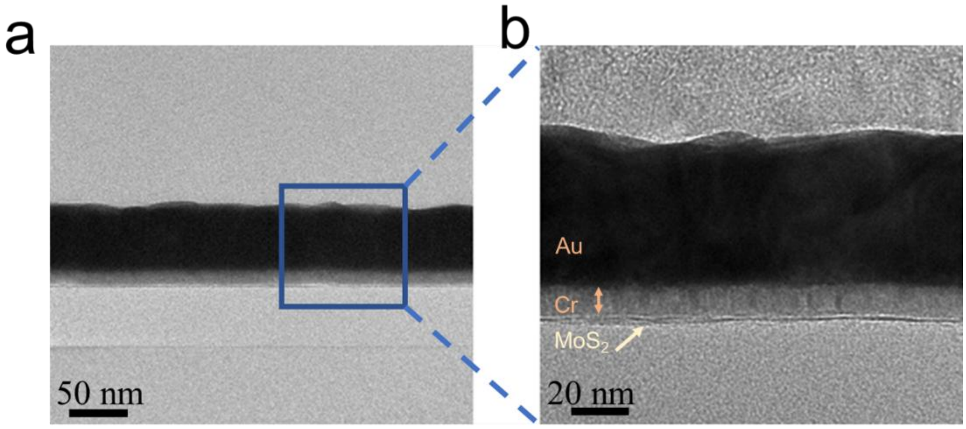

Figure S10 A cross-sectional TEM image of (a) MoS_2/Cr interface after the PLA treatment at PL energy 2.0 mJ (b) A magnified image showed no diffusion for Cr/Au-contacted 1L-MoS_2 FET.

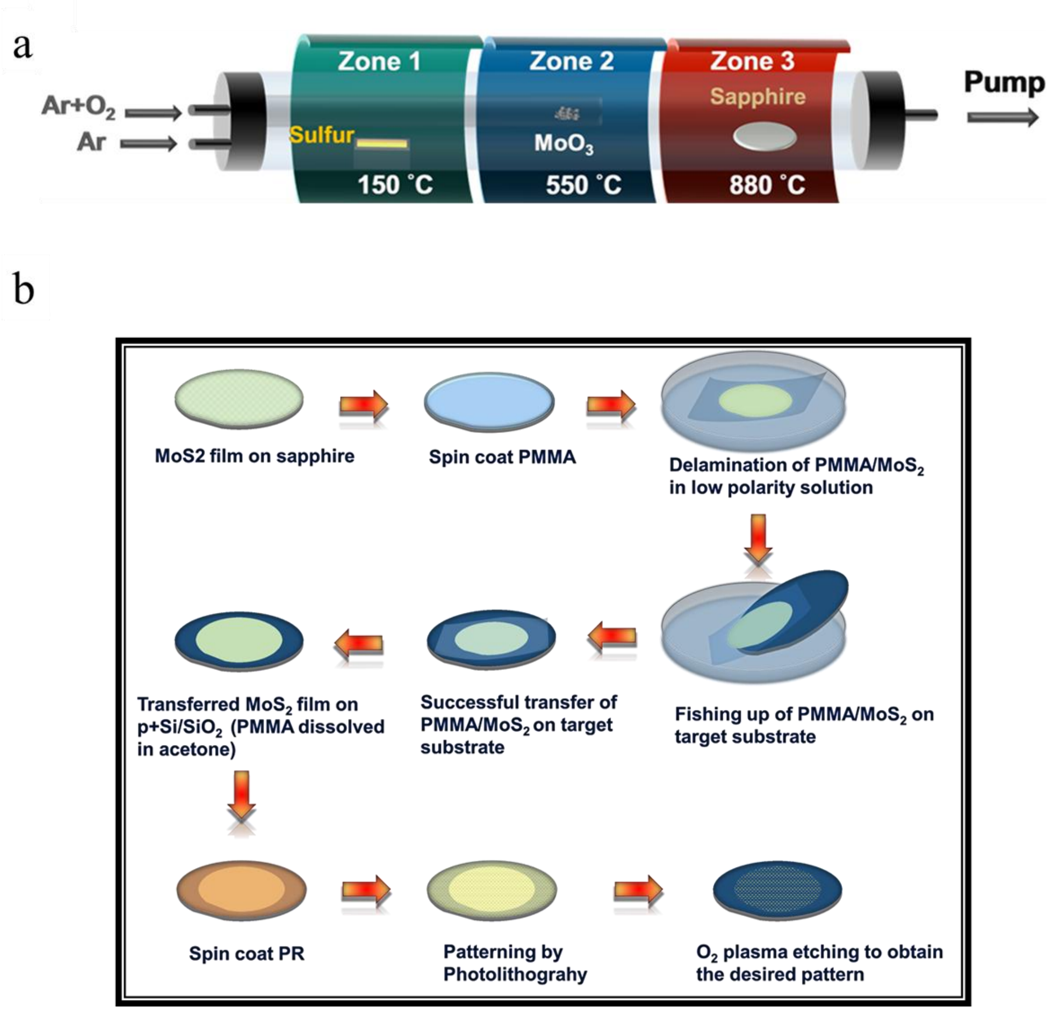

Figure S11 (a) A schematic illustration of the CVD growth process on a 2-inch 1L-MoS_2 thin film. (b) A schematic illustration of the transfer method for the 1L-MoS_2 thin film.
